# geneCBR: a translational tool for multiple-microarray analysis and integrative information retrieval for aiding diagnosis in cancer research

**DOI:** 10.1186/1471-2105-10-187

**Published:** 2009-06-18

**Authors:** Daniel Glez-Peña, Fernando Díaz, Jesús M Hernández, Juan M Corchado, Florentino Fdez-Riverola

**Affiliations:** 1 ESEI: Escuela Superior de Ingeniería Informática, University of Vigo, Edificio Politécnico, Campus Universitario As Lagoas s/n 32004, Ourense, Spain; 2 Computer Science Department, University of Valladolid, Escuela Universitaria de Informática, Plaza Santa Eulalia, 9–11, 40005, Segovia, Spain; 3 Hematological Service, Hospital Universitario de Salamanca and Centro de Investigación del Cáncer (CIC), Universisty of Salamanca-CSIC, Campus Miguel de Unamuno, 37007, Salamanca, Spain; 4 Computer Science Department, University of Salamanca, Plaza de la Merced s/n, 37008, Salamanca, Spain

## Abstract

**Background:**

Bioinformatics and medical informatics are two research fields that serve the needs of different but related communities. Both domains share the common goal of providing new algorithms, methods and technological solutions to biomedical research, and contributing to the treatment and cure of diseases. Although different microarray techniques have been successfully used to investigate useful information for cancer diagnosis at the gene expression level, the true integration of existing methods into day-to-day clinical practice is still a long way off. Within this context, case-based reasoning emerges as a suitable paradigm specially intended for the development of biomedical informatics applications and decision support systems, given the support and collaboration involved in such a translational development. With the goals of removing barriers against multi-disciplinary collaboration and facilitating the dissemination and transfer of knowledge to real practice, case-based reasoning systems have the potential to be applied to translational research mainly because their computational reasoning paradigm is similar to the way clinicians gather, analyze and process information in their own practice of clinical medicine.

**Results:**

In addressing the issue of bridging the existing gap between biomedical researchers and clinicians who work in the domain of cancer diagnosis, prognosis and treatment, we have developed and made accessible a common interactive framework. Our geneCBR system implements a freely available software tool that allows the use of combined techniques that can be applied to gene selection, clustering, knowledge extraction and prediction for aiding diagnosis in cancer research. For biomedical researches, geneCBR *expert mode *offers a core workbench for designing and testing new techniques and experiments. For pathologists or oncologists, geneCBR *diagnostic mode *implements an effective and reliable system that can diagnose cancer subtypes based on the analysis of microarray data using a CBR architecture. For programmers, geneCBR *programming mode *includes an advanced edition module for run-time modification of previous coded techniques.

**Conclusion:**

geneCBR is a new translational tool that can effectively support the integrative work of programmers, biomedical researches and clinicians working together in a common framework. The code is freely available under the GPL license and can be obtained at .

## Background

Recent studies in human cancer have demonstrated that microarrays can be used to develop a new taxonomy of cancer, including major insights into the genesis, progression, prognosis, and response to therapy based on gene expression profiles [1]. However, there continues to be a need to develop new approaches to (*i*) diagnose cancer early in its clinical course, (*ii*) more effectively treat advanced stage diseases, (*iii*) better predict a tumor's response to therapy prior to the actual treatment, and (*iv*) ultimately prevent the appearance of the disease through chemopreventive strategies. Given that systematic classification of tumor types is crucial to achieving advances in cancer treatment, different machine learning and statistical techniques have been successfully applied for cancer classification at the gene expression level. These methods include the successful application of neural networks [2], classification trees and mixture models [3], hierarchical clustering [4], support vector machines [5], shrunken centroids [6,7], compound covariate [8], partial least square [9], principal component analysis disjoint models [10], factor mixture models [11], consensus analysis of multiple classifiers using non-repetitive variables [12], diagonal quadratic discriminant analysis with generalized rule induction [13] etc.

However, while tremendous effort has been invested during recent years in improving the accuracy of novel and existing methods and techniques, minimal effort has been put into developing tools concerned with the application of informatics theory and methods to translational research. As a result, most informatics systems in use today are inadequate in terms of handling the tasks of complicated operations and the management and analysis of contextual data input. In this context, case-based reasoning (CBR) systems are particularly applicable to the domain of translational medicine because they (*i*) support a rich and evolvable representation of experiences/problems, solutions and feedback, (*ii*) provide efficient and flexible ways to retrieve existing data, and (*iii*) can apply analogical reasoning to solve new problems [14]. The research of [15] suggested that analogical reasoning is particularly applicable to the biological domain, partly because biological systems are often homologous (rooted in evolution). Moreover, clinicians often use a form of reasoning similar to CBR, where experiments are designed and performed based on the similarity between features of a new system and those of previously known systems.

In this sense, the research of [16] proposes a mixture of experts for case-based reasoning (MOE4CBR). Previously, [17] showed their initial research in applying a CBR approach to the problem of gene-finding in mammalian DNA. Previously successful research in the same area using CBR was carried out by Shavlik [18]. Lieber and Bresson showed how their CASIMIR/CBR system was able to suggest solutions for breast cancer treatment by adapting the rules of a previous rule-based system [19]. Jurisica and Glasgow demonstrate how case-based reasoning can be applied to assist in analyzing genomic sequences and determining the structure of proteins [14]. They also provide an overview of several other applications in molecular biology that have benefited from CBR.

In this paper we present geneCBR, a translational tool for multiple-microarray analysis and integrative information retrieval for aiding diagnosis in cancer research. The application is intended to be used by three different kinds of users with distinct but related objectives working on the same problem: the systematic classification of tumor types.

## Implementation

**Figure 1:**
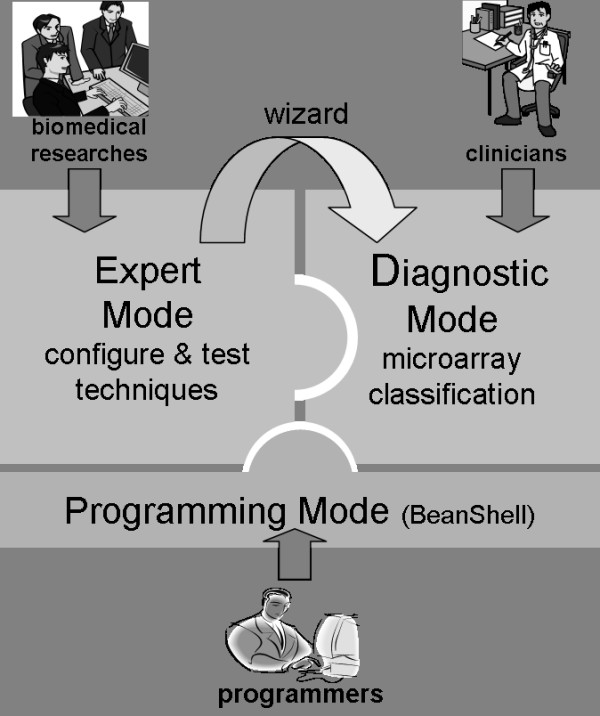
**Translational conception of geneCBR system**. The tree-layer architecture of geneCBR supports the collaborative work of programmers, biomedical researches and clinicians using the same framework.

geneCBR was conceived in order to support integrative work for interdisciplinary research groups working together to design, implement and test new techniques for supervised and unsupervised cancer classification and clustering. Figure 1 shows this user-dependent architecture:

• *Clinicians*: geneCBR (*diagnostic mode*) implements an effective and reliable system for pathologists or oncologists that can diagnose cancer subtypes based on the analysis of microarray data using an underlying CBR architecture.

• *Biomedical researches*: geneCBR (*expert mode*) offers a core workbench for designing and testing new techniques and experiments. It also includes a wizard capable of generating a preconfigured and easy-to-use tool for clinicians.

• *Programmers*: geneCBR (*programming mode*) includes an advanced edition module for run-time modification of previous coded techniques based on BeanShell project .

geneCBR is implemented as a DB-free Java GUI standalone application that can run without any other software requirements (databases, third-party software, etc.) except for a Java Runtime Environment (JRE). Because of the mobility of Java language, the whole system can be launched in different platforms without restraint. The application was tested in both Intel Pentium/Core 2 Duo/Xeon and AMD Athlon CPUs on Windows XP/Vista, Ubuntu Linux 8.04 version and Max OSX 10.5 with Intel architecture and Java 1.5 run-time environment installed.

With regards to the translational nature of geneCBR, it supports three operation modes: *expert *mode, *diagnostic *mode and *programming *mode.

### Expert Mode

**Figure 2:**
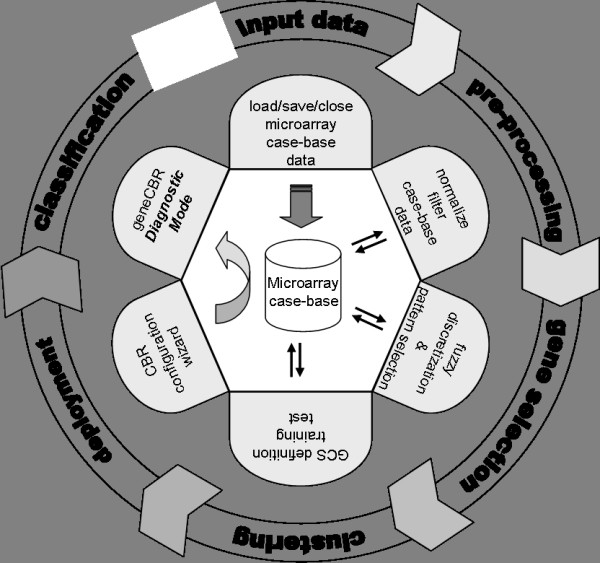
**geneCBR logical processing pipeline and data workflow**. The modular design of the system makes it easy to include new analyses. Furthermore, it is possible to run only specific parts of the pipeline.

Figure 2 shows the logical processing pipeline and data workflow available in geneCBR expert mode. The software exhibits several unique presentations and user-friendly elements by following five simple steps: input data, pre-processing, gene selection, clustering and deployment [see Additional file 1 for a detailed explanation]. Moreover, geneCBR incorporates two advanced modules (Log module and NetExplorer DB Query) that are available during the design, test, and deployment of the preconfigured application for geneCBR diagnostic mode operation.

#### Step 1: Loading microarray data

geneCBR can load microarray gene expression datasets stored in the form of case bases. A case base holds information about the gene values (also called "features") of various patients (also called "exemplars") with a given (or unknown) disease type. These raw data are structured into an open text-based, comma-separated file which also contains meta-data information about each patient (age, sex, karyotype, observations, etc.) [see Additional file 1 for technical documentation].

Each loaded case base is graphically displayed showing both raw data and meta-data information using two available representations: a tabular view and a raw intensities colored view. The application also provides the option of showing only those pathologies of interest.

#### Step 2: Case base pre-processing

After uploading and visualizing the case base, the microarray samples or gene groups to be excluded in later analyses can be filtered from the existing data by creating a new refined case base. The selected exemplars and features can also be saved into a different geneCBR case base file. Additionally, the application provides a built-in operation to normalize gene expression values.

In order to correctly handle the enormous amount of data loaded and processed in steps 1–2, geneCBR implements an advanced internal swapping architecture capable of supporting as many loaded case bases as needed by silently transferring data between memory and massive storage devices.

#### Step 3: Gene selection and noise filtering

geneCBR provides an implementation of a discriminant analysis module (DFP) capable of performing fuzzy codification and studying gene expression raw data [20]. The whole algorithm comprises three main phases involving (*i*) the calculation of different fuzzy membership functions for each gene, (*ii*) the computation of the corresponding discretized gene expression values and (*iii*) the identification of a fuzzy pattern representing the gene expression signature of each disease.

Once the user completes three previous phases, geneCBR constructs a discriminant fuzzy pattern by intersecting gene expression signatures in order to select a final set of discriminative genes. The result from this operation will generate a new case base containing only informative genes (those genes able to discern a specific disease type from others).

#### Step 4: Patient clustering

geneCBR implements an unsupervised clustering module that incorporates a GCS neural network [21] able to group together all patients that are genetically similar according to the case base generated in step 3 [22]. Since such networks contain explicit distance information, they can be used effectively to (*i*) represent an indexing structure which indexes sets of related patients and (*ii*) to serve as a similarity measurement between individual patients. Once the network is trained, geneCBR stores the model which can be tested by using a new case base that incorporates unseen exemplars.

#### Step 5: Deployment of geneCBR diagnostic mode

Every time the biomedical research group finishes its work by configuring, testing and tuning the implemented techniques, the application provides a 4-step guided wizard to setup geneCBR in diagnostic mode [see Additional file 1]. With this configuration, geneCBR is ready to receive a new sample (microarray experiment) and perform all the programmed analysis in a single step, providing the clinician with an adequately substantiated final diagnosis.

#### Advanced plugins: NetExplorer DB Query and Log module

**Figure 3:**
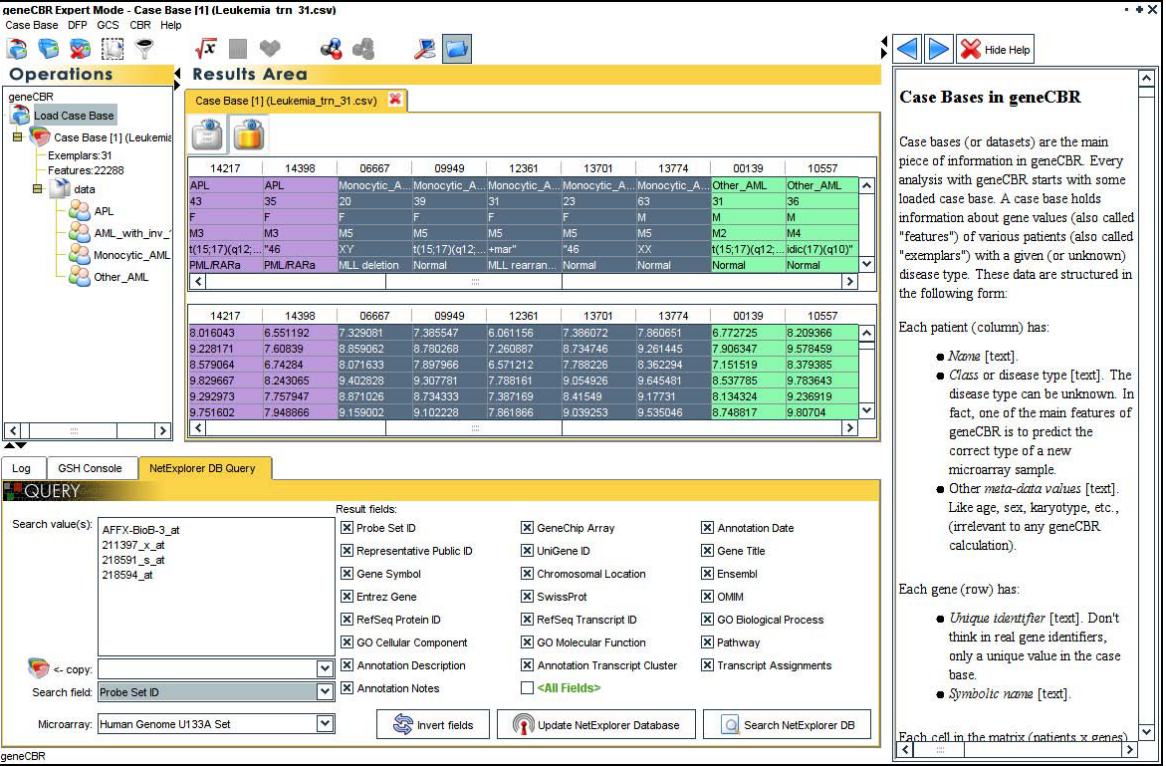
**geneCBR expert mode functionalities**. In expert mode, geneCBR allows biomedical researches by performing multiple-microarray analysis and integrative information retrieval using its implemented techniques. The application also incorporates an extensive context help feature and an easy to follow 4-step wizard to setup geneCBR in diagnostic mode.

The practice of biomedical research seeks to comprehend the intricacy of complex organisms, or their subsystems, by combining many different kinds of data to improve existing knowledge. In addition to the previously commented functionalities, geneCBR includes a *NetExplorer DB Query *module capable of gathering additional information (gene annotations, public gene IDs, biological functions, relevant related articles from PubMed/MedLine, etc.) about interesting gene sets (see Figure 3). This functionality is implemented using geneCBR internal microarray descriptors constructed from the Affymetrix web site [see Additional file 1]. geneCBR always keeps its local files updated by downloading new information as soon as it is available in Internet.

In order to track all the activity carried out by the different techniques implemented in our tool, geneCBR incorporates a *Log *module that always keeps the user updated with relevant information about the results obtained.

### Diagnostic Mode

**Figure 4:**
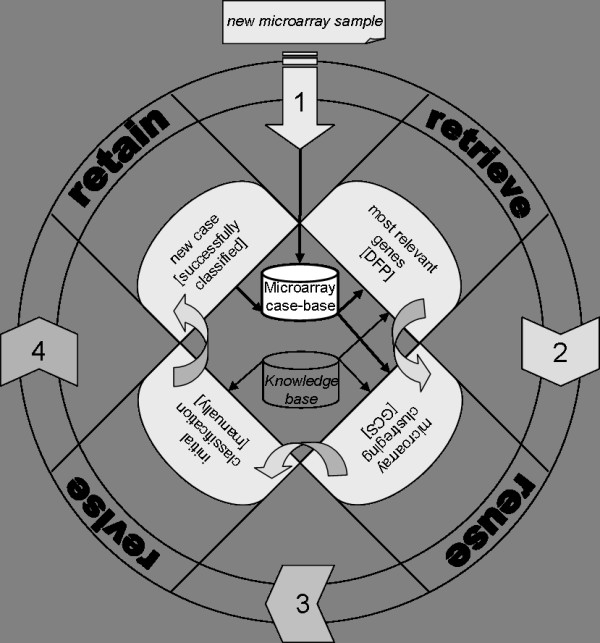
**geneCBR life cycle working in diagnostic mode**. The execution starts by searching the case base and **retrieving **the most informative genes with the new microarray sample to classify. The selected genes are **reused **to train a GCS network for clustering existing patients, and an initial classification is automatically generated. This solution is manually **revised **by the expert and finally, the new case (i.e. the problem description together with the obtained classification) is **retained **in the case base for future use.

Case-based reasoning is a computational reasoning paradigm that involves the storage and retrieval of past experiences to solve new problems. In diagnostic mode, geneCBR employs a previously generated case-based reasoning system that incorporates a discriminant fuzzy pattern for the retrieval of relevant genes, a growing cell structure network for the clustering of similar patients and a proportional weighted voting algorithm to provide an accurate diagnosis. Figure 4 shows the geneCBR life cycle that is automatically launched every time the expert needs to classify a new patient.

While *retrieve *and *reuse *phases (showed in Figure 4) are automatically executed, in the *revise *phase the expert is provided with useful information about the initial classification made by the system and a final decision is required [see Additional file 2]. The information provided by the system contains the selected group genes (DFP), the clustering of patients made by the GCS network, and the weights assigned to each class. The expert contrasts the initial prediction given by the system with other external information like patient karyotype or clinical history in order to ascertain a revised prediction and a final diagnosis. In the *retain *phase, every time a new classification is generated, the knowledge base of geneCBR is updated and the new microarray is associated to its corresponding class and added to the case base.

### Programming Mode

In order to support programmers who work in collaboration with biomedical researches, geneCBR integrates the GSH (geneCBR shell) console available in geneCBR expert mode. The GSH console allows scripting in Java language and supports a wide variety of functionalities including, but not limited to, rapid prototyping, user scripting extension, rules engines, configuration, testing, dynamic deployment, embedded algorithms, etc.

The GSH console is based on the BeanShell project, a small, free, embeddable Java source interpreter with object scripting language features. BeanShell dynamically executes standard Java syntax and extends it with common scripting conveniences such as loose types, commands, and method closures.

**Figure 5:**
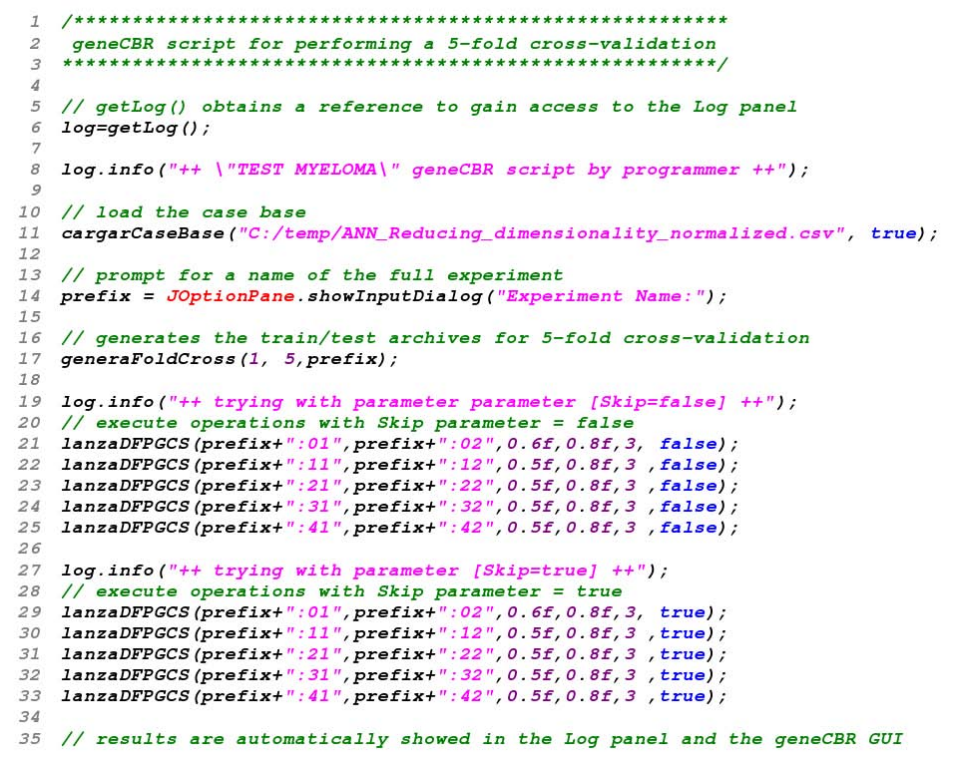
**geneCBR script showing the functionality of the GSH console**. Through the GSH console and the BeanShell project geneCBR gives support to RAD (*Rapid Application Development*).

By utilizing the GSH console, the programmer can easily code new scripts with the goal of preparing and testing novel techniques and experiments provided by the biomedical research group. Once a new functionality is implemented in the GSH console, it can be rapidly integrated in geneCBR expert and diagnostic modes. Figure 5 shows a sample code of a GSH script performing a 5-fold cross-validation.

geneCBR scripts can be directly typed or pasted in the GSH console, or they can be loaded from a file with .gsh extension. The functionality of executing those scripts is only available in geneCBR expert mode.

## Results and Discussion

The concept of translational research has received very strong focus in the biomedical community over the last few years as a new way of thinking about and conducting life sciences research to accelerate healthcare outcomes. However, in order to fully accomplish its objectives, translational research requires clinicians, researchers and the various operational staff to be capable of working together effectively.

Although there are a great number of successful methods and techniques that can be applied to the discovery of cancer subtypes using microarray data [23], none of the implemented tools deals with the translational nature of the problem that we consider essential for the target interdisciplinary research community. For this reason, our geneCBR system is specifically intended to bridge the existing gap between basic research and applied research, and support the collaborative work of biomedical researchers and clinicians by implementing a common framework of interaction. In this respect, geneCBR is, as far as we know, the only application able to aid in the diagnosis of cancer research from this translational perspective.

Moreover, a number of desirable characteristics should be satisfied in order for a particular tool or system to be considered for intensive use in the domain of translational medicine. First, it must be freely available for download and local installation without any restrictions regarding specific agreements, as is the case for geneCBR. Second, it should be based, when possible, on freely-available, open-sourced, successful methods, techniques or methodologies. All the functionalities provided by geneCBR, except for our proposed DFP method, are based on such tools. Third, regarding software memory requirements, an efficient usage of available resources is mandatory. As such, geneCBR implements an advanced internal swapping architecture capable of supporting as many loaded case bases and analyses as needed. Fourth, geneCBR includes all the necessary steps for multiple-microarray analysis with the goal of aiding diagnosis in cancer research, i.e., pre-processing, gene selection, clustering and classification, arranged in a sequential, completely automated pipeline in diagnostic mode. Fifth, with regards to data mining and knowledge extraction, geneCBR offers the NetExplorer DB Query advanced module, which integrates several widely spread standard sources of information, thus making it possible to retrieve relevant information about selected groups of genes.

geneCBR has been tested using several publicly available microarray datasets related to different pathologies of cancer [24,25]. The results provided by geneCBR have confirmed that affected genes published by the original authors are relevant in classifying unknown patients. Several examples can be found and replicated using the sample data and manuals distributed with the geneCBR application.

The geneCBR project also makes available a web site containing valuable information for the research community. The home section introduces the tool and gives information about case-based reasoning systems. In the download section, we have developed multiplatform installers for Windows and Mac/Linux users together with a step-by-step installation guide. Expert mode and diagnostic mode tutorials are also available as separate documents along with sample datasets and a CBR configuration file. The demo section presents several videos about the utilization of different functionalities belonging to the geneCBR logical processing pipeline and data workflow.

## Conclusion

We developed geneCBR with the goal of providing translational support to the integrative work of programmers, biomedical researches and clinicians working together in a common framework. The application implements a set of combined techniques that can be applied to gene selection, clustering, knowledge extraction and prediction for aiding diagnoses in cancer research. geneCBR offers a set of core modules and features that are not currently available in other biomedical decision support systems. The application is written entirely in Java 1.5 and is portable across multiple operating systems and platforms. No hardware or memory restrictions are imposed by this software. It is well documented and simple to execute through the utilization of the provided installation wizard [see Additional file 3].

Although the numerical analysis of microarray data is considerably consolidated, the true integration of numerical analysis and biological knowledge is still a long way off [26]. The future of geneCBR involves the inclusion of additional knowledge sources in the classification process. This functionality can prevent the discovery of the obvious data-inferred hypothesis that references previously proposed relationships, and through its analysis help avoid overconfident predictions, thus allowing experts to systematically relate the analysis findings to present knowledge [27]. In this regard, our goal is to pave the way for the principled integration of imperfect biological knowledge with gene expression data and other high-throughput data sources in order to make predictions that are easy to interpret in concert with incorporated knowledge.

In summary, we consider this bioinformatic tool as an open and evolving project. The application is free, as it has been released under the GPL license, and its development is open and collaborative. Researchers are free to use it, to modify it, and to deploy their own web site with the results.

## Availability and requirements

**Project name**: geneCBR

**Project home page**: 

**Operating systems**: Platform independent

**Programming language**: J2SE 1.5

**Other requirements**: none

**License**: GNU GPL

## Competing interests

The authors declare that they have no competing interests.

## Authors' contributions

FD and JMC designed the translational architecture and the knowledge base. DGP implemented geneCBR application and programmed the web site. JMH and JMC tested geneCBR as end-users and wrote the supplementary material. FFR wrote the paper while DGP, FD, JMH and JMC provided comments and discussion. FFR guided and coordinated the development of geneCBR. All authors read and approved the final manuscript.

## Supplementary Material

Additional file 1 **Expert Mode manual**. This document will guide the user through a step-by-step tutorial showing the capabilities of geneCBR to setup and save an optimized configuration that can automatically classify new samples in *Diagnostic Mode*. Click here for file

Additional file 2 **Diagnostic Mode manual**. This document will guide the user through a step by step tutorial showing the capabilities of geneCBR to automatically classify new microarray samples in *Diagnostic Mode*. Click here for file

Additional file 3 **Installation guide**. This manual covers the installation of geneCBR and its related components through the utilization of an installation wizard. Click here for file
