# Supplementary material for: geneCBR: a translational tool for multiple-microarray analysis and integrative information retrieval for aiding diagnosis in cancer research
Source: BMC Bioinformatics. 2009 Jun 18;10:187. doi: 10.1186/1471-2105-10-187 (PMC2703634; doi:10.1186/1471-2105-10-187)
Supplement: Additional file 1 — Expert Mode manual. This document will guide the user through a step-by-step tutorial showing the capabilities of geneCBR to setup and save an optimized configuration that can automatically classify new samples in Diagnostic Mode. [file 1471-2105-10-187-S1.pdf]

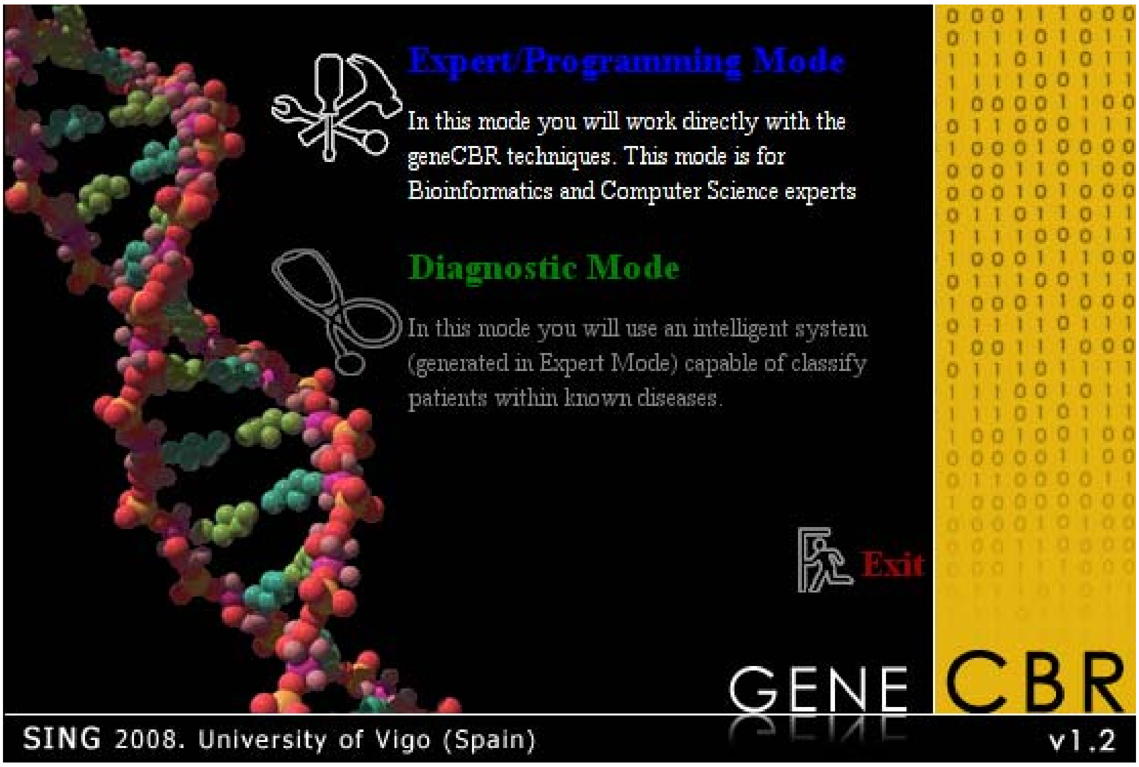

GENECBR

Expert Mode Manual

This document will guide you through a step by step tutorial showing the capabilities of GENECBR to setup and save an optimized configuration able to automatically classify new samples in Diagnostic Mode.

Contents

Case Bases in GENECBR 1
Welcome to GENECBR 3
Case Base menu 4
  Loading a case base 4
  Saving a case base 5
  Normalize data 6
  Filtering genes and/or samples 7
  Closing a case base 8
DFP menu 10
  Calculate Membership Functions 10
  Calculate Fuzzy Discretization 11
  Calculate Fuzzy Patterns 14
GCS menu 18
  Create GCS Network 18
  Test GCS Network 19
CBR menu 23
  Create CBR 23
  Load CBR 27
Help menu 28
  Update NetExplorer Database 28
  GENECBR Help 30
  Visit www.genecbr.org 30
Advanced modules 32
  Log module 32
  GSH Console 32
  NetExplorer DB Query 32
Exiting GENECBR 34
Bibliography 35

Case Bases in GENECBR

Case bases (or datasets) are the main piece of information in GENECBR. Every analysis with GENECBR starts with some loaded case base. A case base holds information about gene values (also called "features") of various patients (also called "exemplars") with a given (or unknown) disease type. These data are structured in the following form:

Each patient (column) has:

- *Name* [text].
- *Class* or disease type [text]. The disease type can be unknown. In fact, one of the main features of GENECBR is to predict the correct type of a new microarray sample.
- Other *meta-data values* [text]. Like age, sex, karyotype, etc., (irrelevant to any GENECBR calculation).

Each gene (row) has:

- *Unique identifier* [text]. Don't think in real gene identifiers, only a unique value in the case base.
- *Symbolic name* [text].

Each cell in the matrix (patients x genes) has:

- *Expression value* of each gene [decimal number].

Internally, GENECBR works with text-based, comma-separated files (like csv) to load/save case bases. These files must be in a specified, but simple, format. A GENECBR case base file has the following format:

The first line contains:

- First column: "UNIQID" [text, different in all lines].
- Second column: "NAME" [text].
- Other columns: patient names.

The "Class" line holds the disease type of each patient:

- First column: "#" [text].
- Second columns: "Class" [text].
- Other columns: patient's disease name [text]. If the disease type is unknown, it stores a blank space.

Metadata lines: holding human readable meta-data about patients.

- First column: sharp character (#).
- Second column: meta-data's name [text]. For example age, sex, karyotype, etc.
- Other columns: values of this meta-data variable for each patient [text].

Other lines:

- First column: a gene identifier [text]. GENECBR does not use any namespace like NCBI gene IDs. You can put here any, but unique, ID.
- Second column: a gene name [text].

- Other columns: gene expression values for each patient, one column per sample [decimal number, the decimal separator is a dot (.)].

Here is an example of this file:

```
UNIQID,NAME,05204,00185,06667,00139,10557
#,Class,APL,AML_with_inv_16,Monocytic_AML,Other_AML,Other_AML
#,Age,38,32,20,31,36
#,Sex,F,M,F,M,M
#,FAB/WHOa,M3,M4Eo,M5,M2,M4
#,FISH studiesb,PML/RARa,CBFB/MYH11,MLL deletion,Normal,Normal
1,AFFX-BioB-5_at,6.694213,6.336728,7.329081,6.772725,8.209366
2,AFFX-BioB-M_at,7.805106,7.540492,8.859062,7.906347,9.578459
3,AFFX-BioB-3_at,6.827084,6.975979,8.071633,7.151519,8.379385
…
22283,222384_at,3.754731,3.746064,4.008511,3.806199,4.116638
```

In order to correctly follow this step-by-step tutorial, GENECBR is now distributed with sample microarray data collected from Gene Expression Omnibus and stored as several GENECBR case base files. Details about the original dataset can be found in

Gutiérrez,NC. López-Pérez,R. Hernández,JM. Isidro,I. González,B. Delgado,M. Fermiñán,E. García,JL. Vázquez,L. González,M. San Miguel,JF. (2005) Gene expression profile reveals deregulation of genes with relevant functions in the different subclasses of acute myeloid leukemia. *Leukemia*. **19(3)**, 402-9.

The Leukemia dataset contains bone marrow samples from 43 adult patients with newly de novo diagnosed AML. All samples contained more than 80% blast cells. The median age was 36 years (range 14-70 years). Patients were classified according to the WHO classification into 4 subgroups: (*i*) 10 APL with t(15;17) confirmed by FISH studies with LSI PML/RARA probe (Vysis, Stuttgart, Germany), (*ii*) 4 AML with inv(16) confirmed by FISH analysis with LSI CBFB probe (Vysis); (*iii*) 7 acute monocytic leukemias and (*iv*) 22 non-monocytic AML without recurrent cytogenetic translocations. Each microarray experiment stores 22,283 expressed sequence tags (ESTs) corresponding to the expression level of thousands of genes measured using Affymetrix - GeneChip® Human Genome U133A.

Based on the previous commented dataset, GENECBR contains the following case base sample files:

`Leukemia_full_43.csv`	Original dataset in GENECBR format.
`Leukemia_trn_31.csv`	31 samples from existing pathologies for training purposes in GENECBR *Expert Mode operation*.
`Leukemia_test_12.csv`	12 samples from existing pathologies for test purposes in GENECBR *Expert Mode operation*.
`Leukemia_test_01.csv`	1 sample for test purposes in GENECBR *Diagnostic Mode operation* (see Diagnostic Mode manual).

Welcome to GENECBR

The welcome screen provides an entry-point and interface to the GENECBR system. If you are a new user, probably you want to go to the GENECBR help or visit the application portal on Internet.

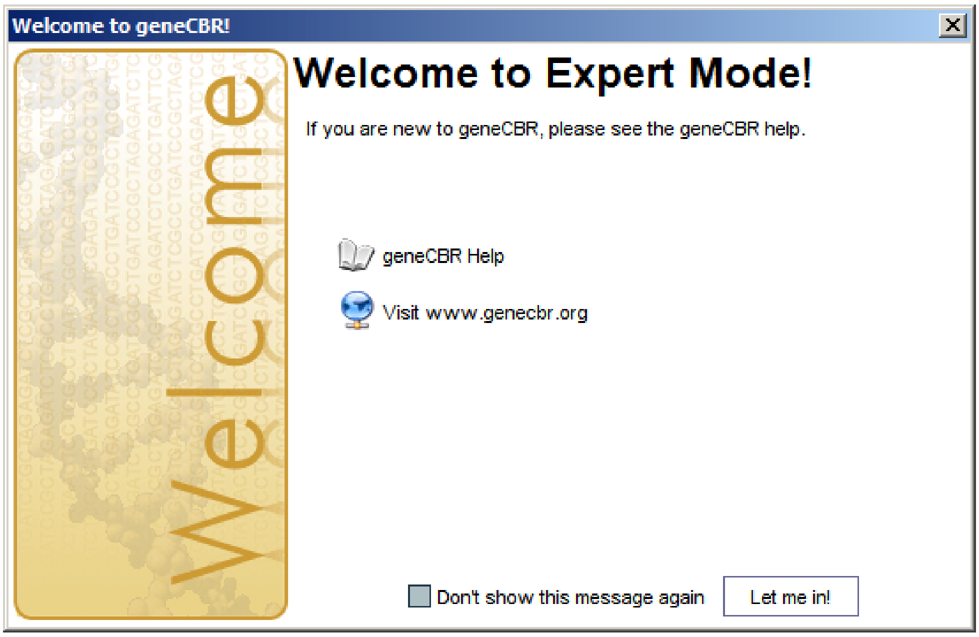

Once you are familiar with the tool, you can get up and running faster by disabling the welcome screen in the bottom of the dialog box.

Case Base menu

Loading a case base

Note: be sure of selecting a text file in the GENECBR file format. Otherwise you will get an error during the load process.

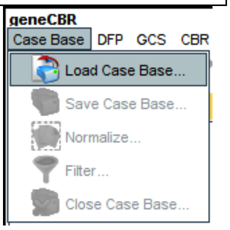

To load a case base from a GENECBR case base file you have to go to the `Case Base->Load Case Base...` menu and select a file in the file chooser dialog

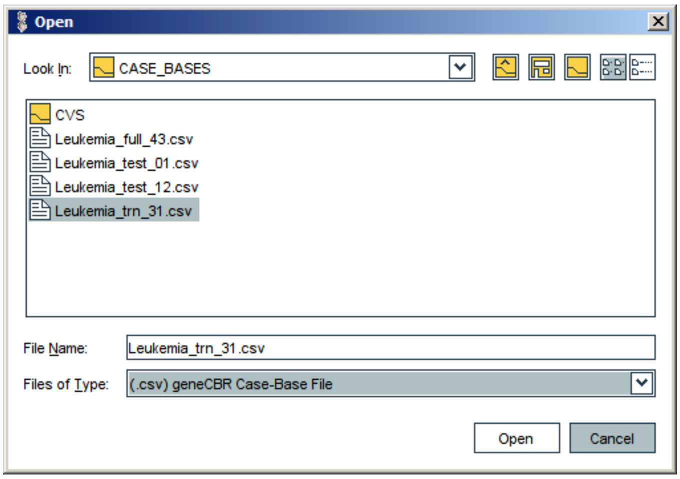

Next, you will see a progress dialog bar while the case base is loading. If some mistake is detected in the case base file, you will get an error during the load process.

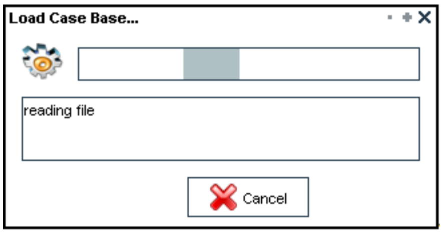

Finally, you will be prompted for a name to assign to the new case base.

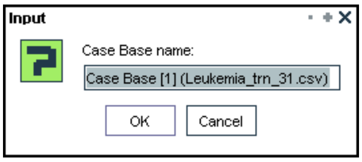

As a result, you will see the loaded case base in both the `Operations` tree (left) and the `Results Area` (right).

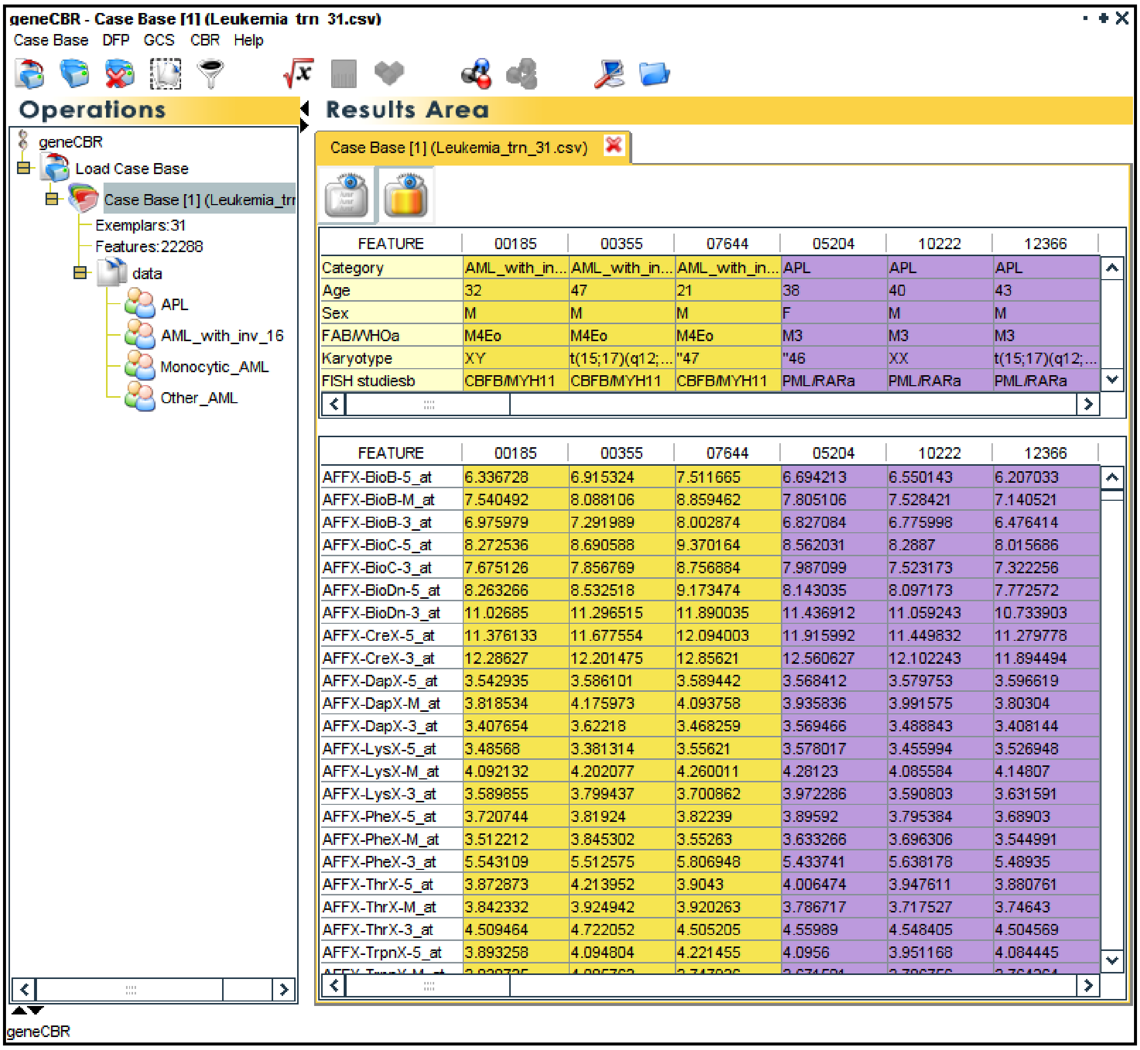

The tabular view (right) shows a textual representation of the case base data. There are two tables: one for the meta-data information provided (up) and another for the gene expression values (bottom).

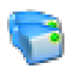

Saving a case base

Note: by default, GENECBR stores the case base in the installation path directory. Be sure you select the correct path.

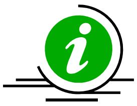

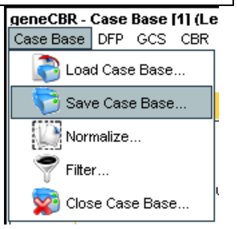

To backup a case base you have to go to the `Case Base->Save Case Base...` menu and select the case base you want to save.

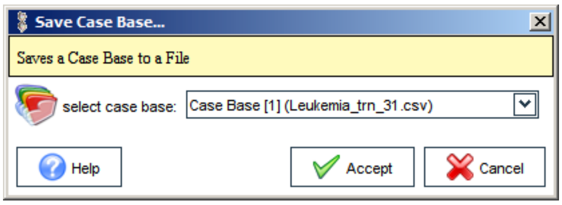

Then, you have to provide a destination filename.

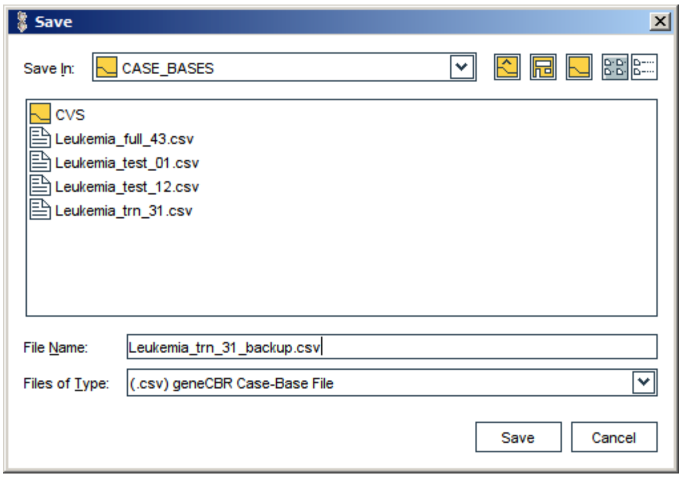

During the backup process you will see a progress dialog bar.

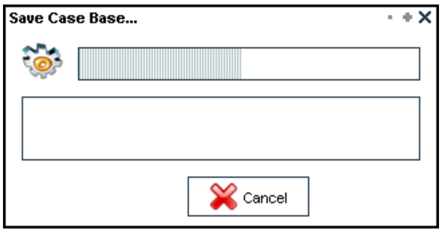

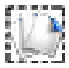

Normalize data

Note: The normalize operation produces a new case base leaving intact the original one.

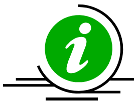

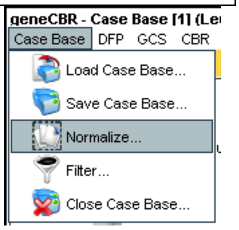

To normalize gene expression data between two given values you have to go to the `Case Base->Normalize...` menu, specify the case base you want to normalize and indicate the min/Max threshold values.

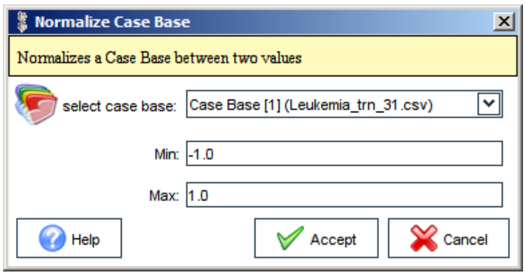

Next, you will see a progress dialog bar meanwhile the normalization process is executed.

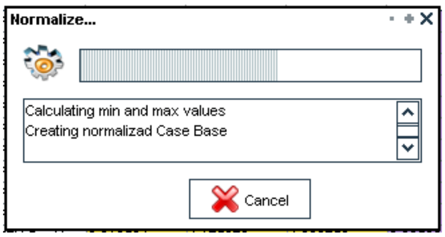

Finally, you will be prompted for a name to assign to the new case base.

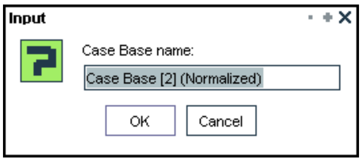

As a result, you will see the normalized case base in both the `Operations` tree and the `Results Area`.

Filtering genes and/or samples

Note: GENECBR can select genes and/or patients to produce a new case base. The filter operation produces a new case base leaving intact the original one.

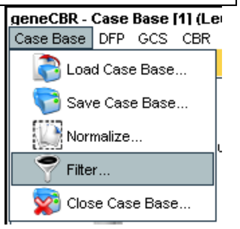

To filter genes and/or patients you have to go to the `Case Base->Filter...` menu, specify the case base you want to filter and multiple select genes and/or patients.

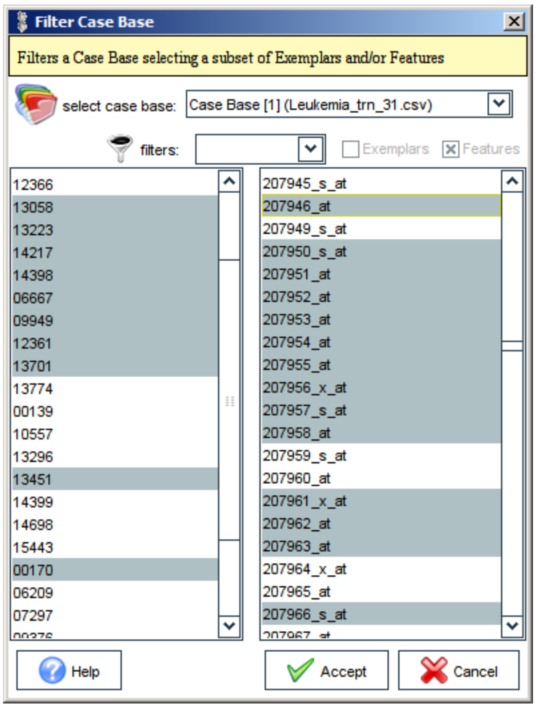

Next, you will see a progress dialog bar meanwhile the filter process is executed.

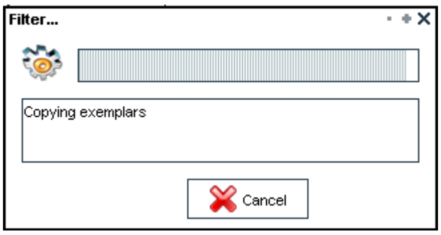

If you did not specify a name for the filter you have executed, GENECBR will prompt you for a name.

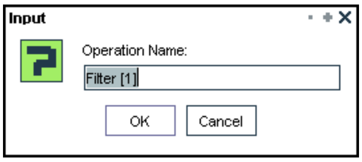

The new filtered case base needs a name, so you have to provide it using the following dialog.

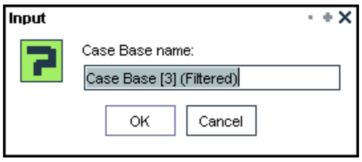

As a result, you will see the filtered case base in both the `Operations` tree and the `Results Area`.

Closing a case base

Note: You can free memory in GENECBR by closing unused case bases.

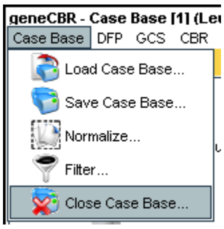

To close an open case base you have to go to the `Case Base->Close Case Base...` menu and specify the case base you want to close.

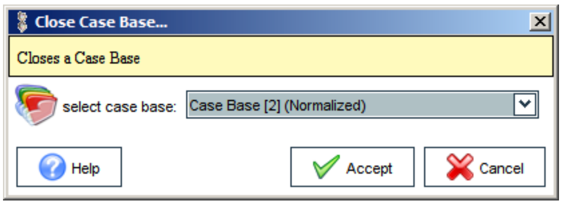

It will disappear from both the `Operations` tree and the `Results Area`.

DFP menu

Calculate Membership Functions

Note: by default, GENECBR defines three linguistic labels (LOW, MEDIUM & HIGH) but you can personalize this functionality.

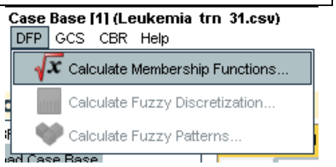

To automatically calculate the membership function for each gene you have to go to the `DFP->Calculate Membership Functions...` menu. In the input dialog you can select the source case base, the number of membership functions you want to use and tick the check box if you want to skip odd gene expression values.

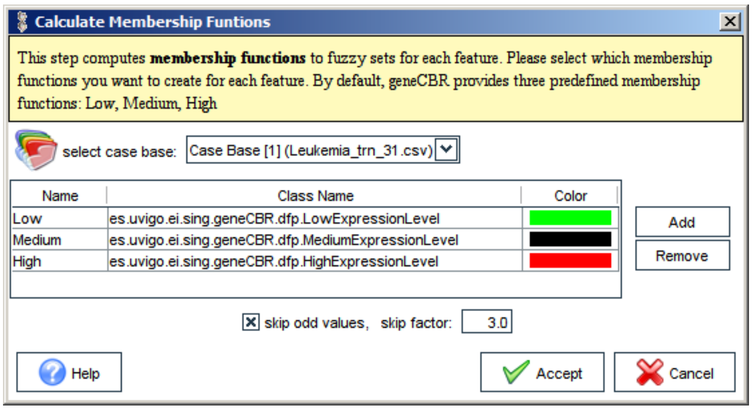

Next, you will see a progress dialog bar meanwhile membership functions are calculated.

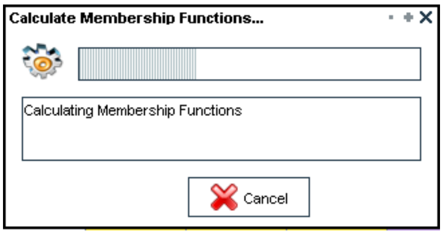

As a result, you will see the membership functions in the `Results Area`. You can select multiple genes and graphically view the shape of its membership functions. Moreover, you can activate the `grid by default` and `exemplars by default` options in order to represent in the same figure the existing patients ordered by their gene expression values.

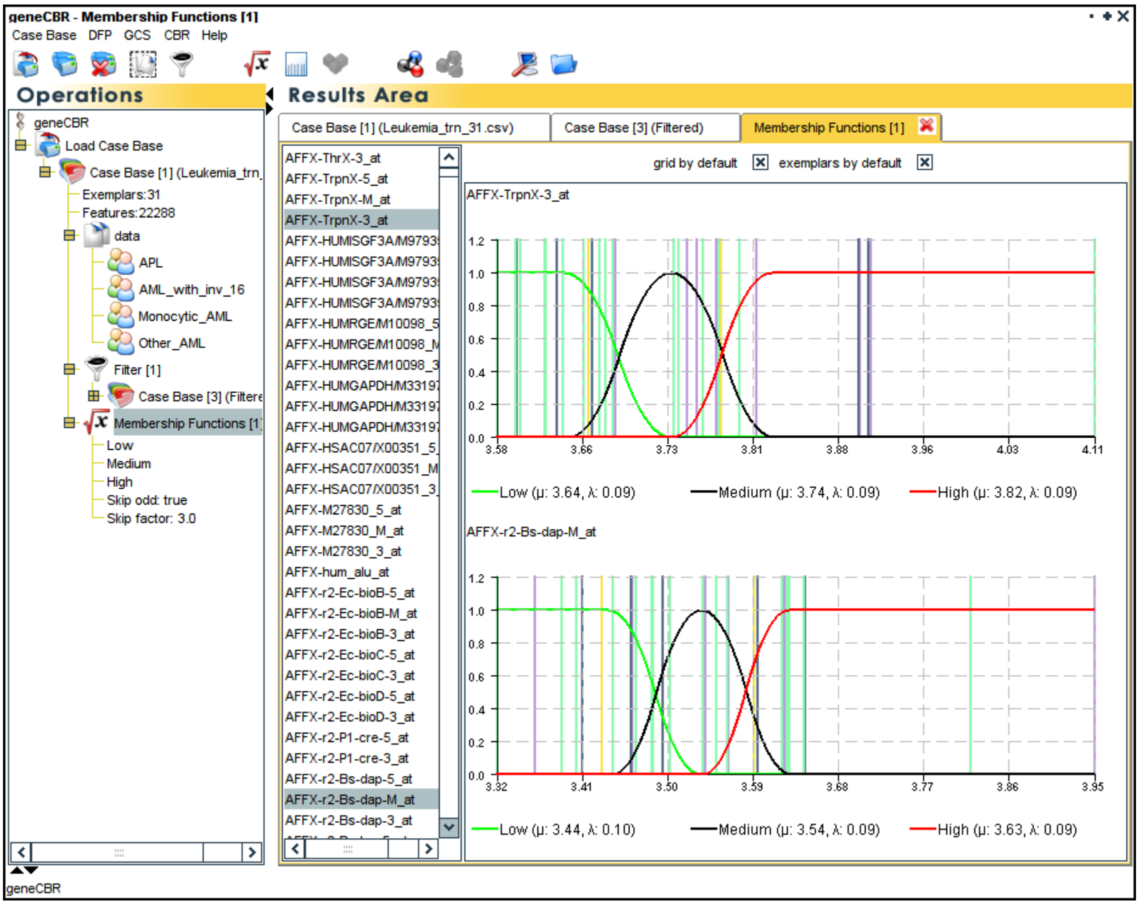

Calculate Fuzzy Discretization

Note: by default, GENECBR defines two overlaps between each linguistic label different from LOW & HIGH, but you can personalize this functionality.

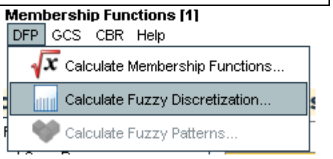

To automatically assign a discretized linguistic label for each gene you have to go to the `DFP->Calculate Fuzzy Discretization...` menu. In the input dialog you can select the source case base, a set of membership functions previously calculated, the level of overlap between membership functions and a threshold (θ value) for assigning a given label to a specific gene expression value.

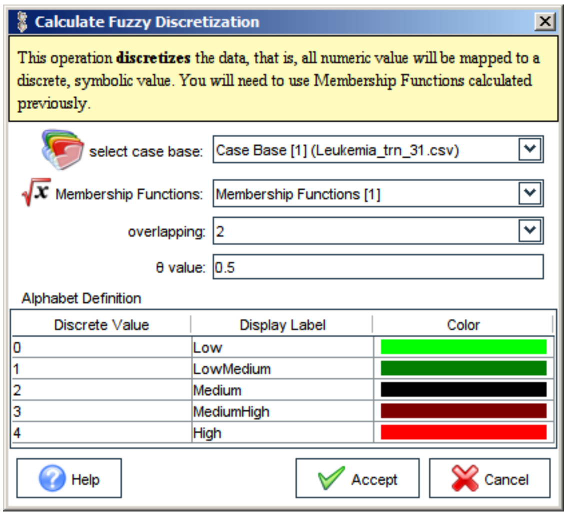

Next, you will see a progress dialog bar meanwhile the discretization process is executed.

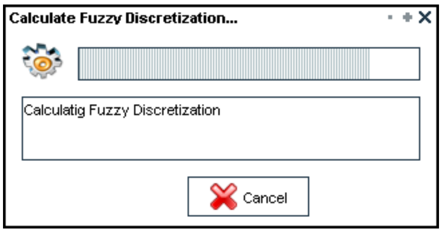

Once the process is terminated, you have to assign a name to the new discretized case base.

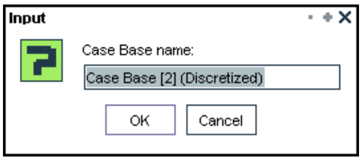

Finally, you will see the new generated case base in both the `Operations` tree (left) and the `Results Area` (right).

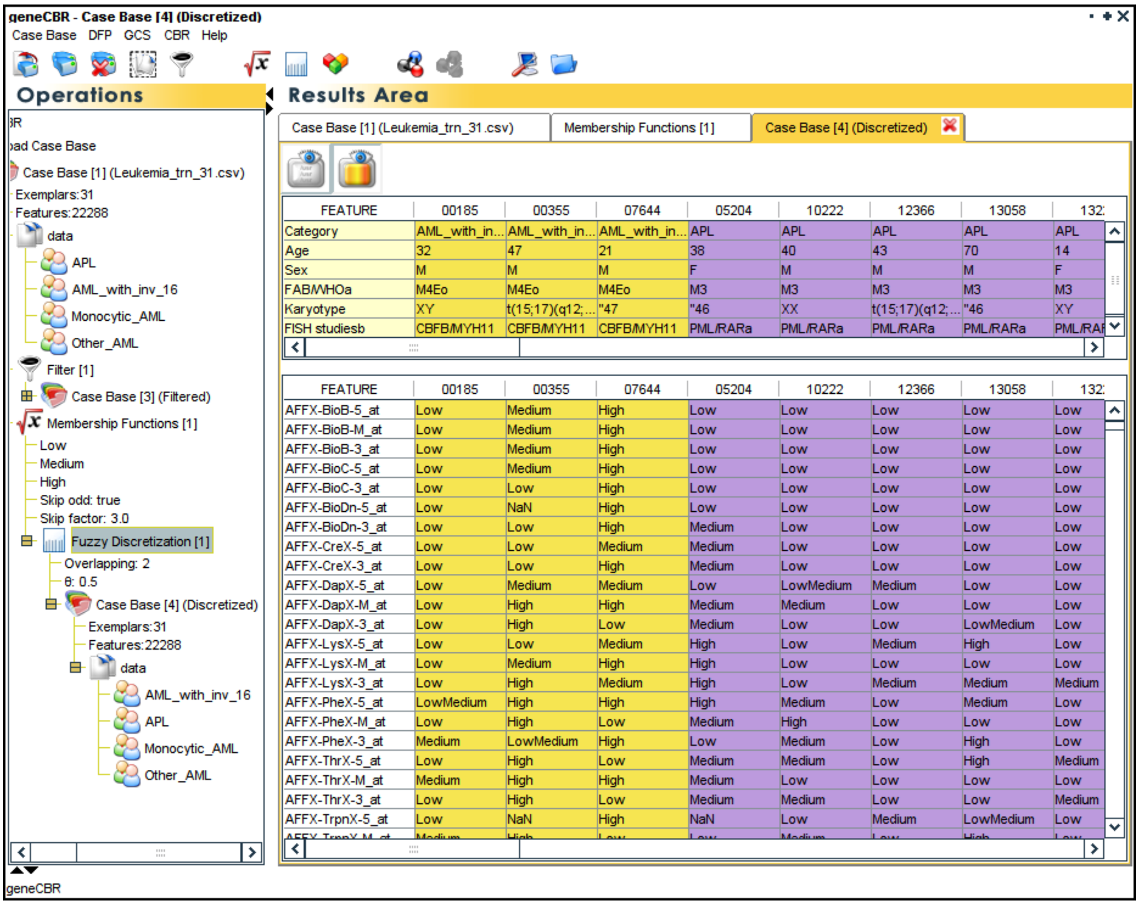

Every time you visualize a case base in the `Results Area`, you can choose between two alternative views of the same data: (i) *raw mode* () and (ii) *colored mode* (). If you select the colored mode, a progress dialog bar is showed while min. and max. gene expression values are calculated.

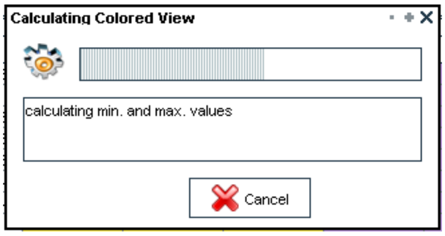

In a few seconds the colored view is renderized and showed in the `Results Area`.

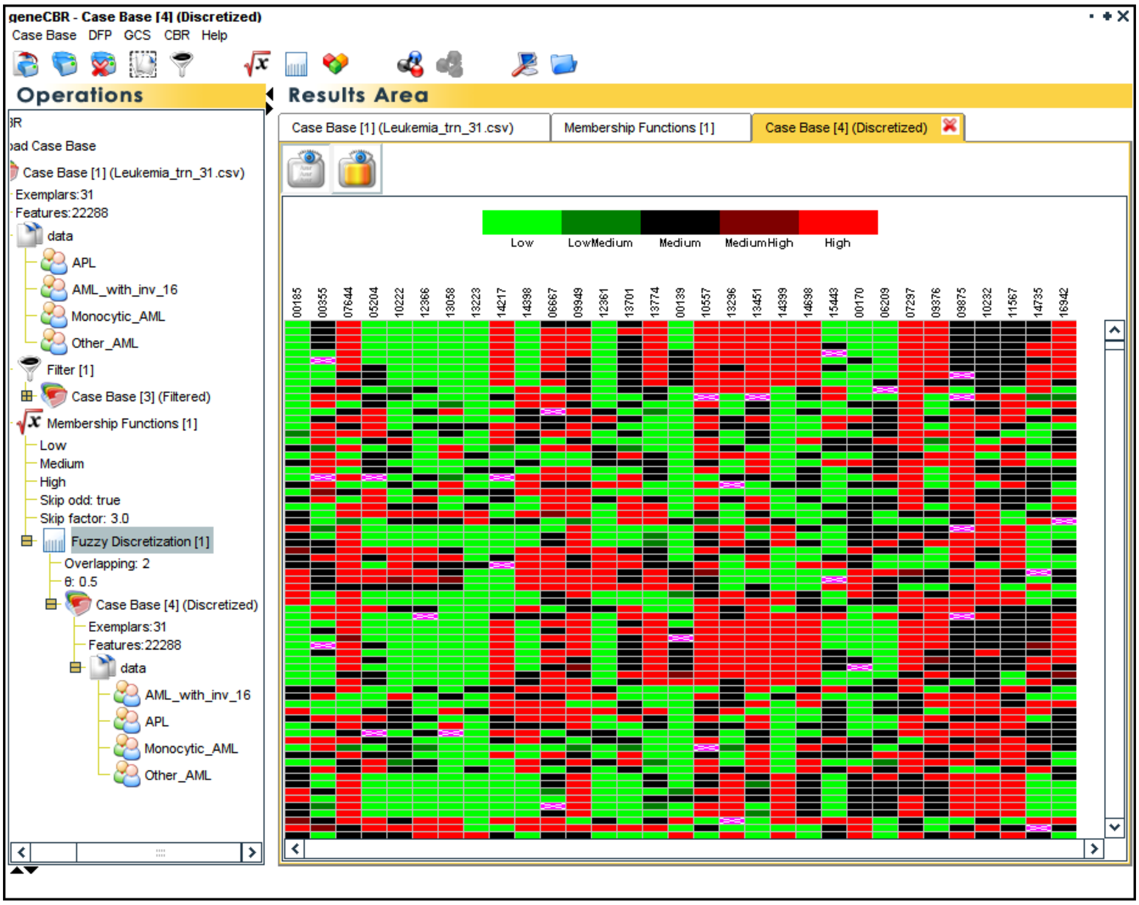

Calculate Fuzzy Patterns

Note: Different fuzzy patterns can be obtained by changing the π parameter that controls the percentage of occurrence of a given linguistic label in samples belonging to the same disease.

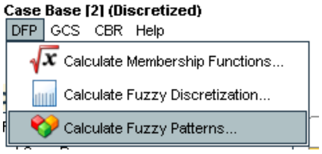

To automatically select those genes that best summarize a given disease you have to go to the `DFP->Calculate Fuzzy Patterns...` menu. In the input dialog you can select the source case base, a fuzzy discretization previously calculated and a threshold (π value) for assigning a given gene to the fuzzy pattern of a disease.

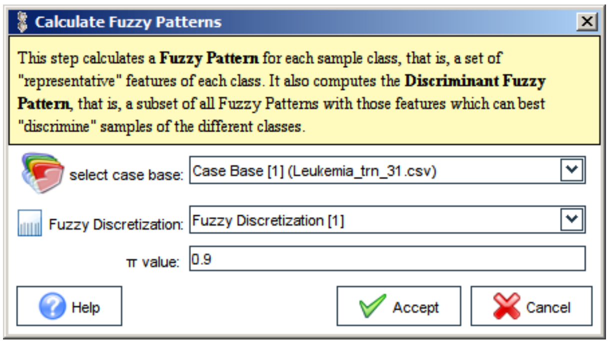

Next, you will see a progress dialog bar meanwhile the fuzzy pattern construction process is executed.

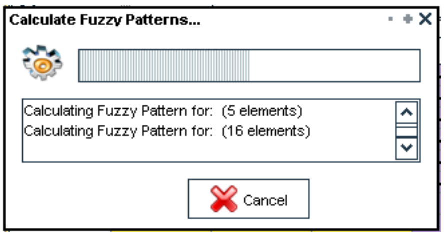

As a result, you will see in the `Results Area` the selected genes for each disease (bottom) and a brief summary of the linguistic labels present in each fuzzy pattern (up). In the `Operations` tree (left) you can see the number of genes that form the discriminant fuzzy pattern (in our example, only 198 genes from the 22288 of an HGU133A Affymetrix array).

In the lower part of the `Results Area` you can now select the type of view you want: (i) showing all genes belonging to some fuzzy pattern or (ii) showing only those genes belonging to the discriminant fuzzy pattern (those genes with a different linguistic label assigned to a different fuzzy pattern).

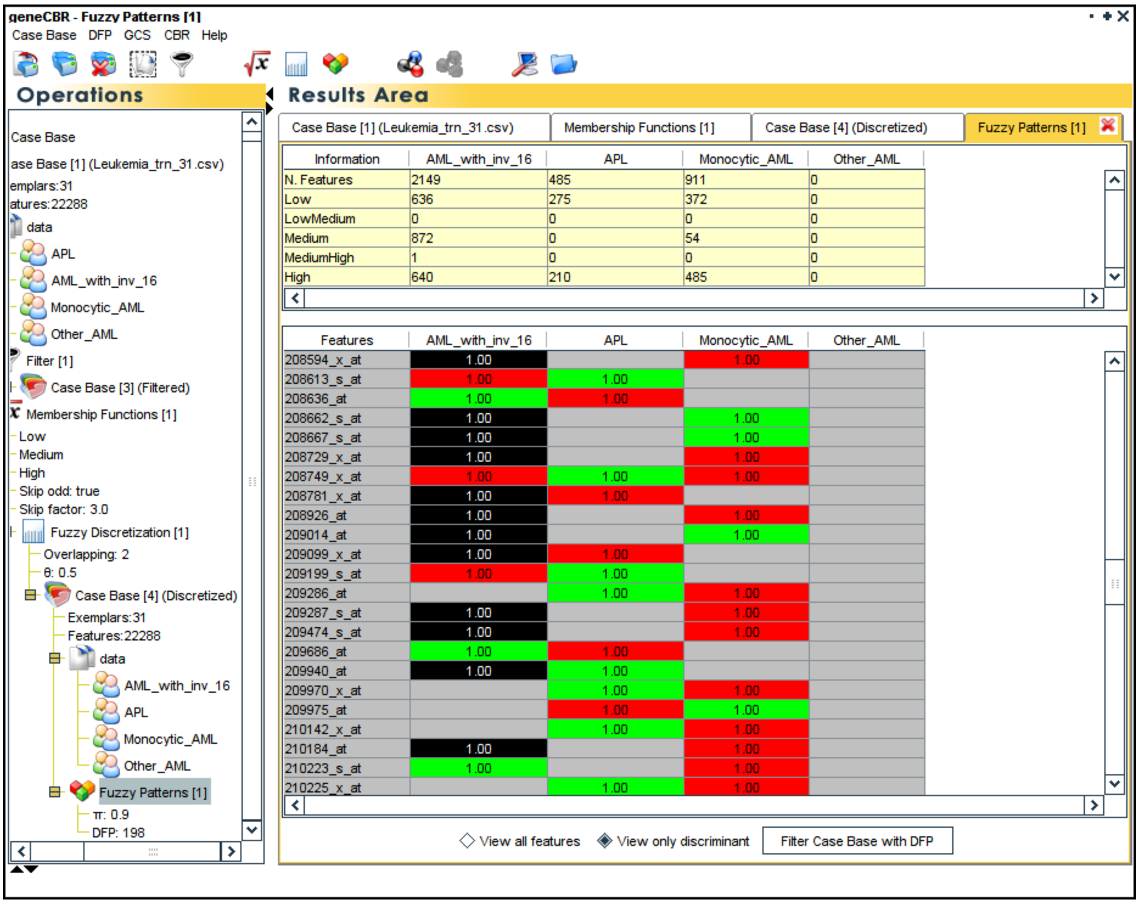

Once a discriminant fuzzy pattern (DFP) is calculated, you are able to filter the original case base using those genes belonging to the DFP. To perform this operation press the `Filter Case Base with DFP` button in the lower part of the `Results Area`.

A new input dialog appears like in the case of the `Case Base->Filter...` menu. By default, those genes belonging to the DFP are selected, so the only thing you need to do is specify the case base you want to filter (in our example, the original one).

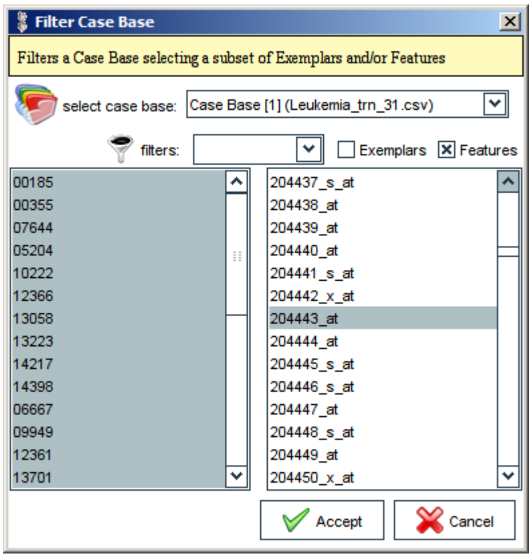

If you did not specify a name for the filter you have executed, GENECBR will prompt you for a name.

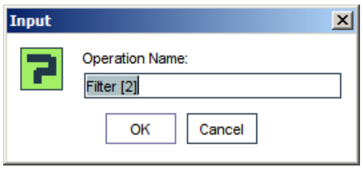

The new filtered case base needs a name, so you have to provide it using the following dialog.

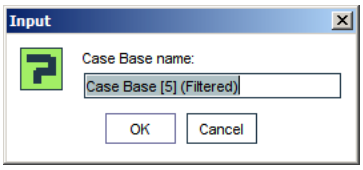

As a result, you will see the DFP-filtered case base in both the `Operations` tree (left) and the `Results Area` (right). In our example, this case base holds the same patients and their meta-data information as in the original case base.

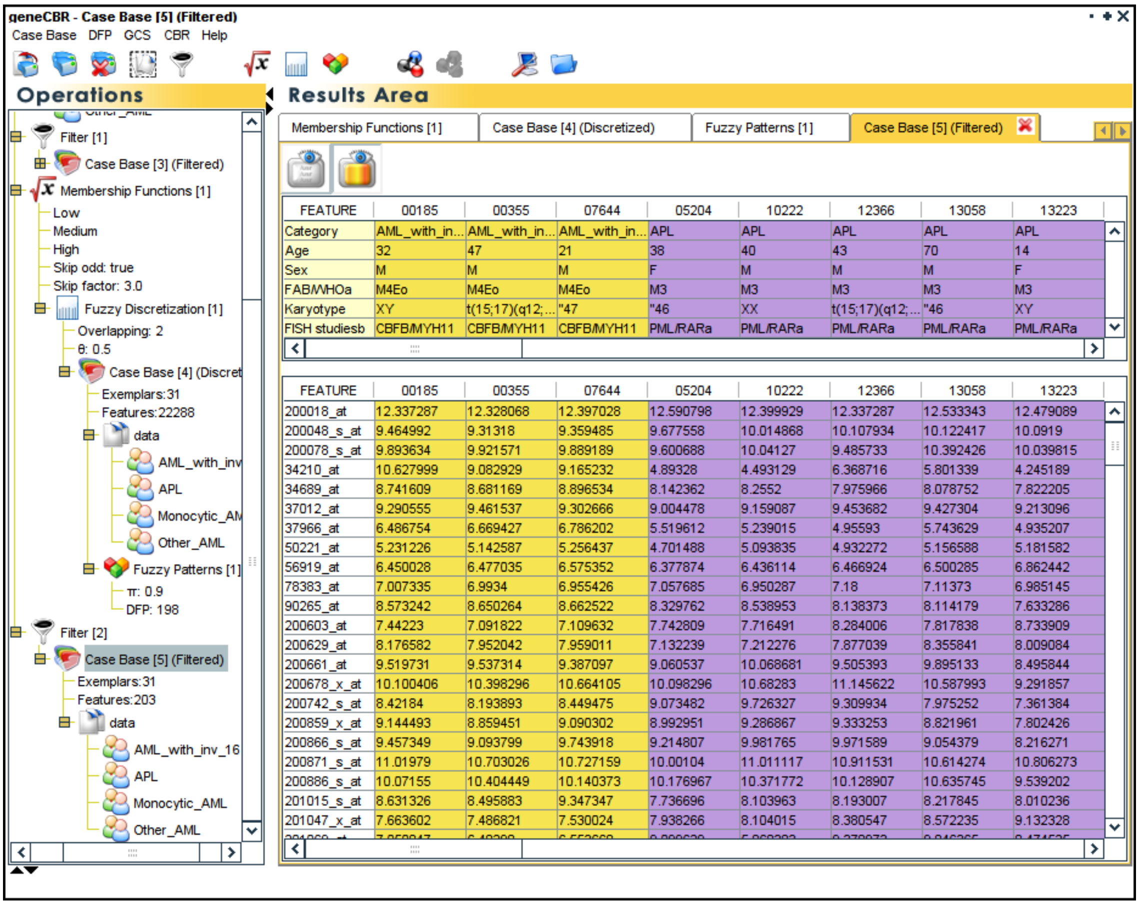
geneCBR - Case Base [5] (Filtered)
Case Base DFP GCS CBR Help
Operations
Filter [1]
Case Base [3] (Filtered)
Membership Functions [1]
Low
Medium
High
Skip odd: true
Skip factor: 3.0
Fuzzy Discretization [1]
Overlapping: 2
θ: 0.5
Case Base [4] (Discret
Exemplars:31
Features:22288
data
AML_with_inv
APL
Monocytic_AM
Other_AML
Fuzzy Patterns [1]
π: 0.9
DFP: 198
Filter [2]
Case Base [5] (Filtered)
Exemplars:31
Features:203
data
AML_with_inv_16
APL
Monocytic_AML
Other_AML
Results Area
Membership Functions [1]
Case Base [4] (Discretized)
Fuzzy Patterns [1]
Case Base [5] (Filtered)
FEATURE 00185 00355 07644 05204 10222 12366 13058 13223
Category AML_with_in... AML_with_in... AML_with_in... APL APL APL APL APL
Age 32 47 21 38 40 43 70 14
Sex M M M F M M M F
FAB/WHOa M4Eo M4Eo M4Eo M3 M3 M3 M3 M3
Karyotype XY t(15;17)(q12;... "47 "46 XX t(15;17)(q12;... "46 XY
FISH studiesb CBFB/MYH11 CBFB/MYH11 CBFB/MYH11 PML/RARa PML/RARa PML/RARa PML/RARa PML/RARa
FEATURE 00185 00355 07644 05204 10222 12366 13058 13223
200018_at 12.337287 12.328068 12.397028 12.590798 12.399929 12.337287 12.533343 12.479089
200048_s_at 9.464992 9.31318 9.359485 9.677558 10.014868 10.107934 10.122417 10.0919
200078_s_at 9.893634 9.921571 9.889189 9.600688 10.04127 9.485733 10.392426 10.039815
34210_at 10.627999 9.082929 9.165232 4.89328 4.493129 6.368716 5.801339 4.245189
34689_at 8.741609 8.681169 8.896534 8.142362 8.2552 7.975966 8.078752 7.822205
37012_at 9.290555 9.461537 9.302666 9.004478 9.159087 9.453682 9.427304 9.213096
37966_at 6.486754 6.669427 6.786202 5.519612 5.239015 4.95593 5.743629 4.935207
50221_at 5.231226 5.142587 5.256437 4.701488 5.093835 4.932272 5.156588 5.181582
56919_at 6.450028 6.477035 6.575352 6.377874 6.436114 6.466924 6.500285 6.862442
78383_at 7.007335 6.9934 6.955426 7.057685 6.950287 7.18 7.11373 6.985145
90265_at 8.573242 8.650264 8.662522 8.329762 8.538953 8.138373 8.114179 7.633286
200603_at 7.44223 7.091822 7.109632 7.742809 7.716491 8.284006 7.817838 8.733909
200629_at 8.176582 7.952042 7.959011 7.132239 7.212276 7.877039 8.355841 8.009084
200661_at 9.519731 9.537314 9.387097 9.060537 10.068681 9.505393 9.895133 8.495844
200678_x_at 10.100406 10.398296 10.664105 10.098296 10.68283 11.145622 10.587993 9.291857
200742_s_at 8.42184 8.193893 8.449475 9.073482 9.726327 9.309934 7.975252 7.361384
200859_x_at 9.144493 8.859451 9.090302 8.992951 9.286867 9.333253 8.821961 7.802426
200866_s_at 9.457349 9.093799 9.743918 9.214807 9.981765 9.971589 9.054379 8.216271
200871_s_at 11.01979 10.703026 10.727159 10.00104 11.011117 10.911531 10.614274 10.806273
200886_s_at 10.07155 10.404449 10.140373 10.176967 10.371772 10.128907 10.635745 9.539202
201015_s_at 8.631326 8.495883 9.347347 7.736696 8.103963 8.193007 8.217845 8.010236
201047_x_at 7.663602 7.486821 7.530024 7.938266 8.104015 8.380547 8.572235 9.132328

GCS menu

Create GCS Network

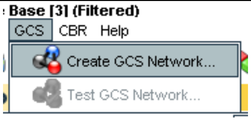

Note: With this option you can create and train a Growing Cell Structures network to test how informative are the genes that form a discriminant fuzzy pattern.

Starting from the previous DFP-filtered case base you can create and train a GCS network for unsupervised patient clustering. To do this you have to go to the `GCS->Create GCS Network...` menu. In the input dialog you can select the source case base, the different parameters governing the GCS learning cycle and the maximum number or runs.

For a simple GCS operation the parameters provided by default are adequate. However, the maximum number of network nodes (`Max. Nodes`) should be established *a priori*.

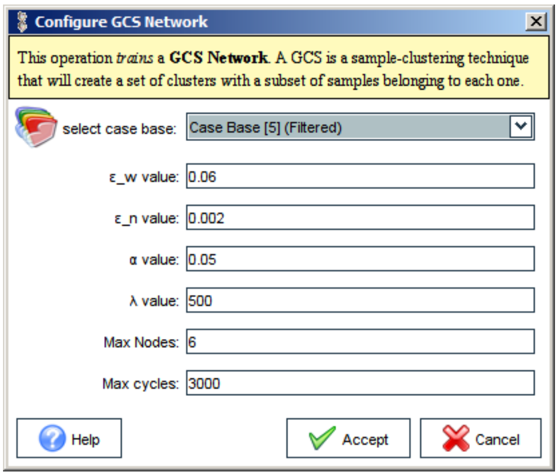

Next, you will see a progress dialog bar meanwhile the learning process is executed.

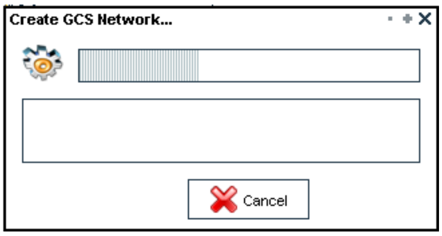

Once the GCS network is trained, you have to assign a name to the new model.

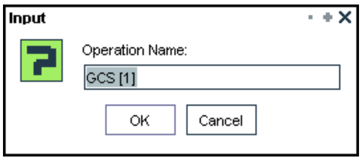

As a result, you will see the network information in both the `Operations` tree (left) and the `Results Area` (right). In our example, the network has six nodes clusterizing all the patients present in the training case base.

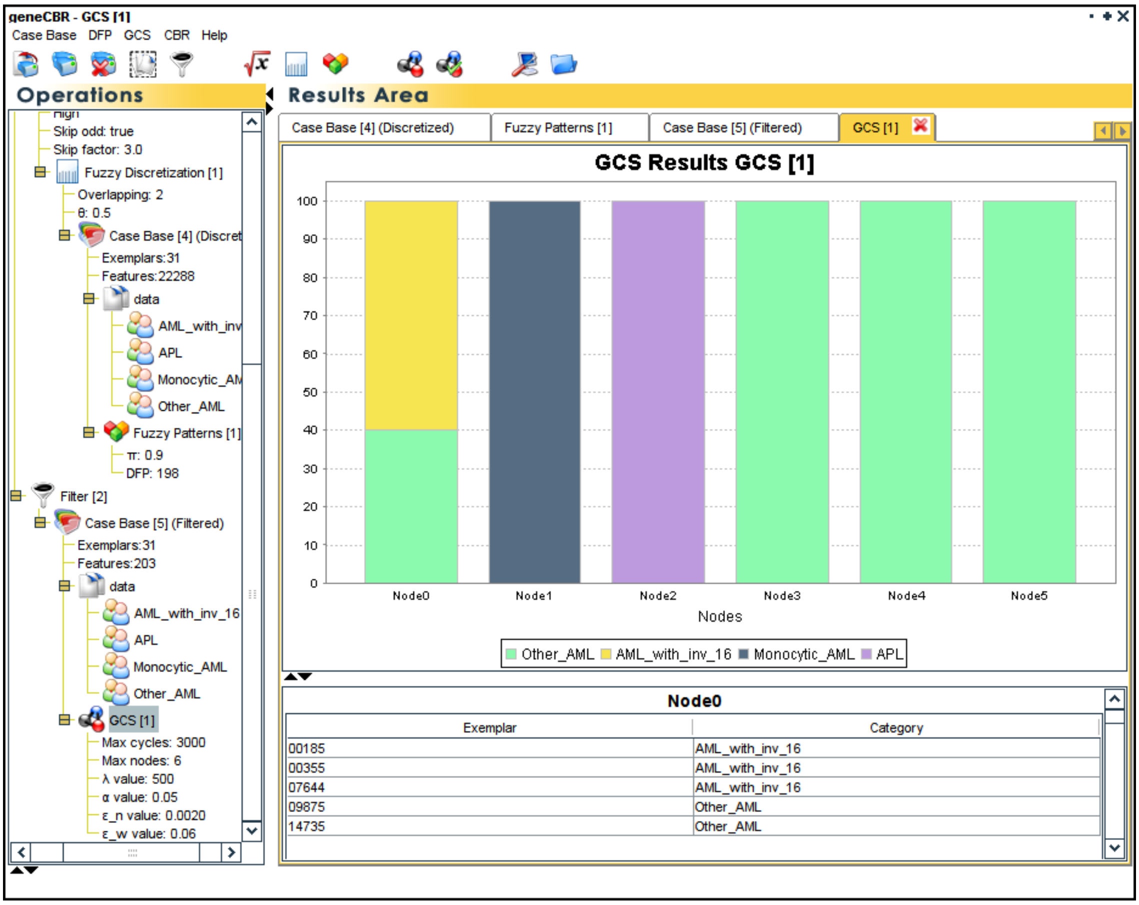

In the lower part of the `Results Area` you can see those patients belonging to each network node.

Test GCS Network

Note: In order to test a previous trained GCS network you need to load and filter a new case base.

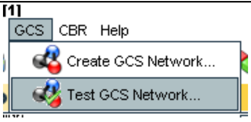

In our example, you have to follow the previously explained procedure for `Loading a case base` in order to load the *Leukemia_test_12.csv* GENECBR case base file. Once you have finished the process you will see the new case base in the `Results Area`.

Take into account that the color assigned to all the patients are the same because we do not know the class of those patients (This information is available in the *Leukemia_full_43.csv* GENECBR case base file).

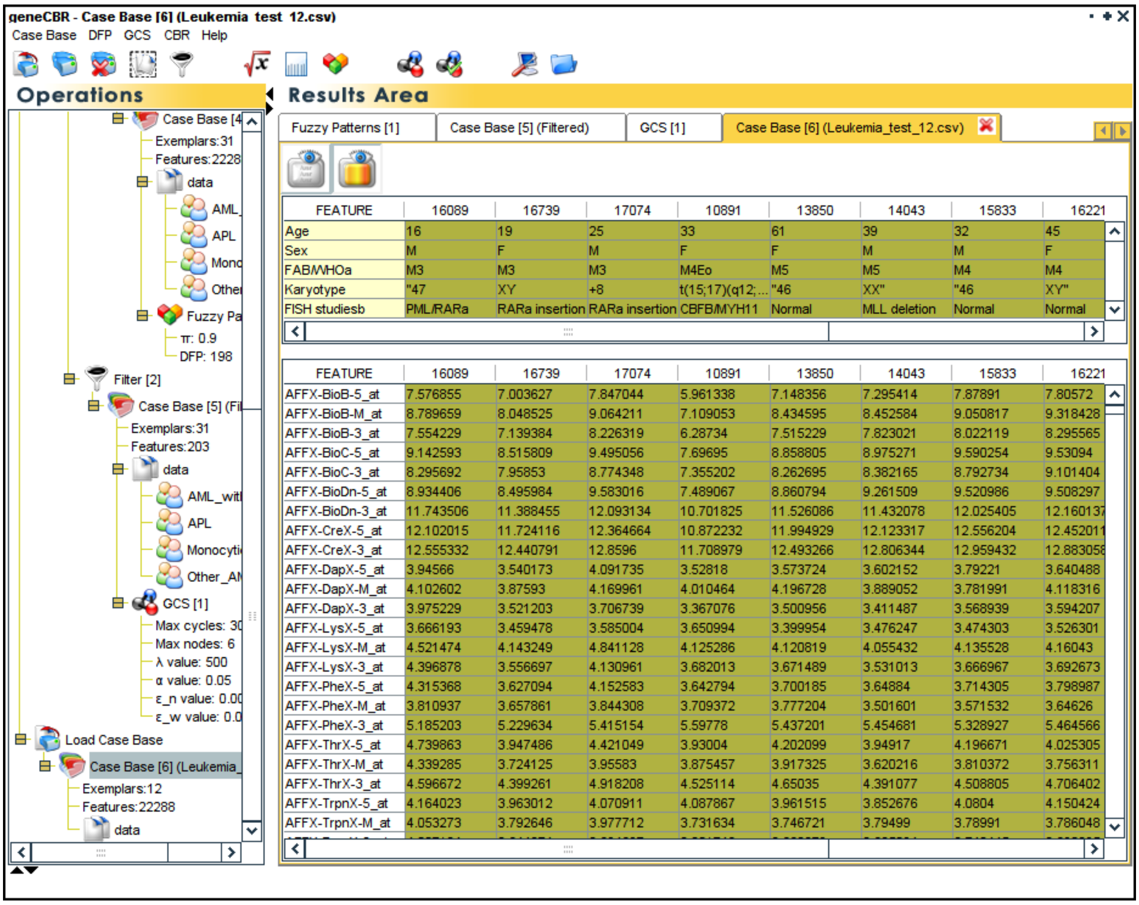

A previous step to test the GCS network is to filter the new loaded case base with those genes belonging to the DFP. To do this you have to follow the previously explained procedure for `Filtering genes and/or samples`.

In the filter input dialog you have to specify the previously loaded case base (*Leukemia_test_12.csv*) and select the filter with name `Filter [2]`.

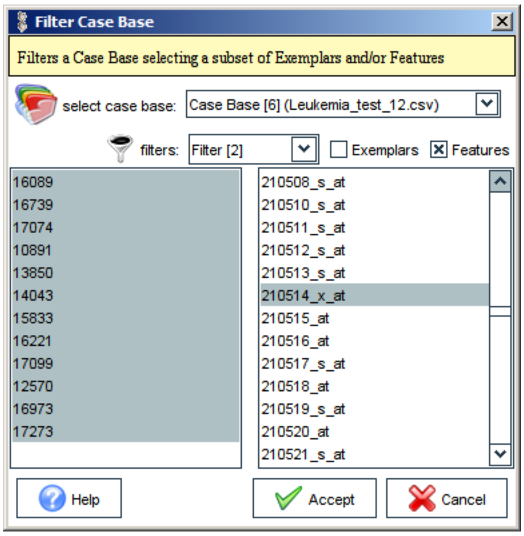

As a result and after specifying a name for the new case base, you will see the filtered case base in both the `Operations` tree and the `Results Area`.

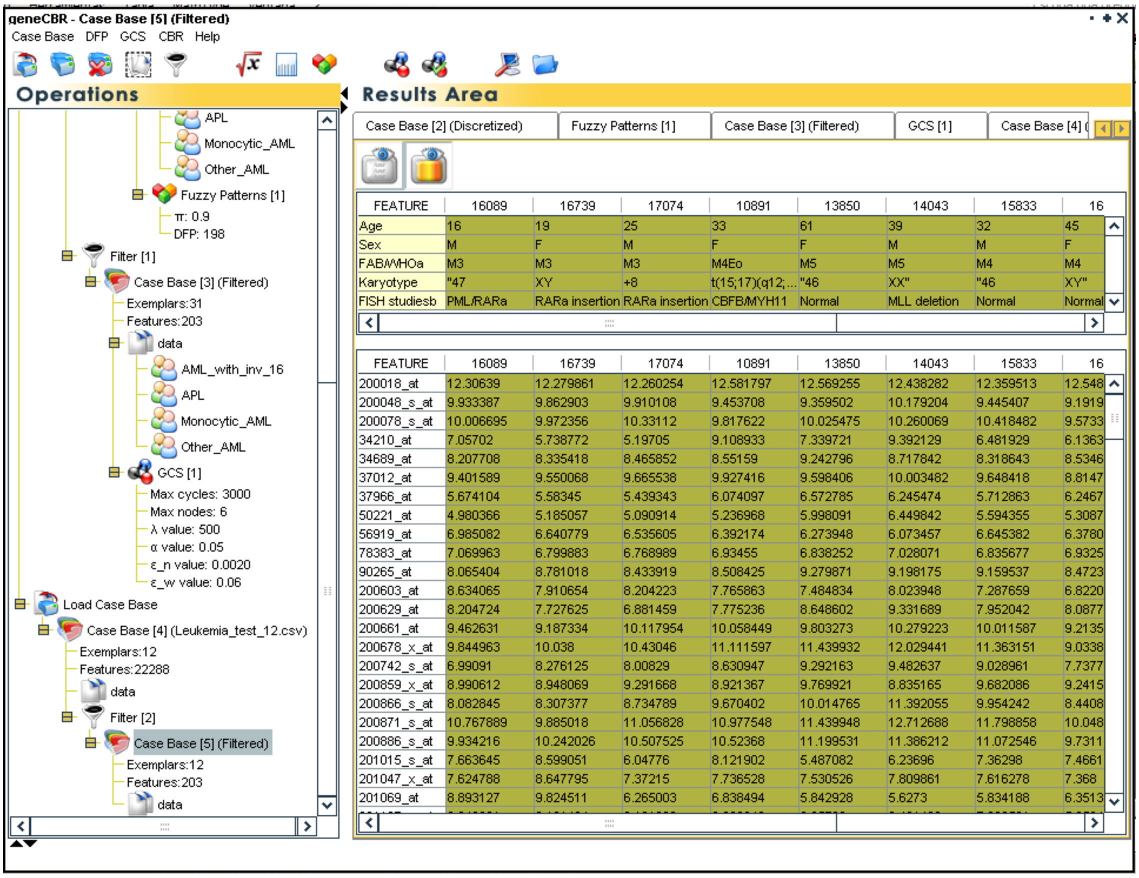

Now, you have all the required information to test the trained network. To do this you have to go to the `GCS->Test GCS Network...` menu, select the trained GCS network and specify the new filtered case base.

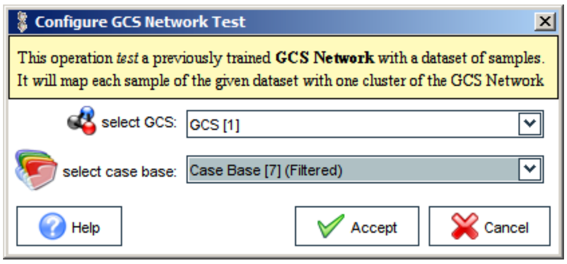

As a result, you will see the network information in both the `Operations` tree (left) and the `Results Area` (right). In our example, the network has clusterized all the patients present in the test case base.

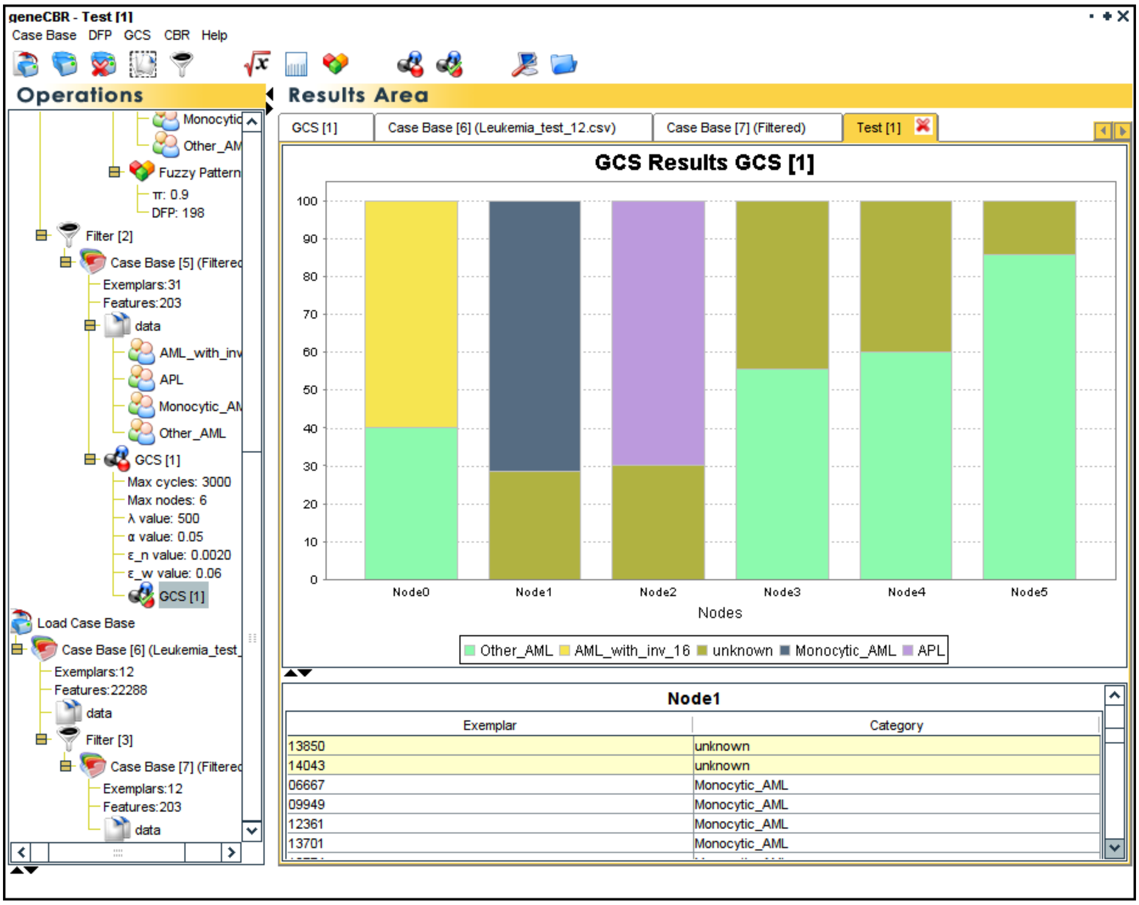

In the lower part of the `Results Area` you can see those patients belonging to each network node. The patients with a solid line are those belonging to the test case base.

CBR menu

Create CBR

Note: This operation allows the expert to setup a preconfigured application able to automatically classify new incoming microarrays samples (with unknown class).

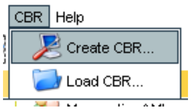

By executing the `CBR->Create CBR...` menu option, a wizard with 4 simple steps is showed to the user. In each stage of the wizard you can go one step forward or go back using the predefined buttons.

The first step involves the creation of the main GENECBR case base file through the specification of a case base containing all the known samples. You can take it from a csv file, or from a previous loaded case base in GENECBR.

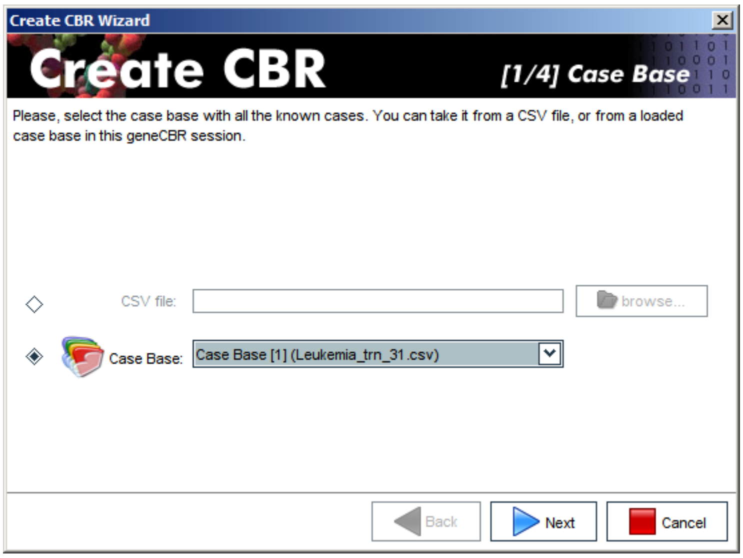

The second step involves three subparts: (i) definition of the membership functions, (ii) configuring the fuzzy discretization process and (iii) establishing the parameters for the construction of the discriminant fuzzy pattern.

In the following screen you can reuse previously defined membership functions or specify a new configuration for their calculation.

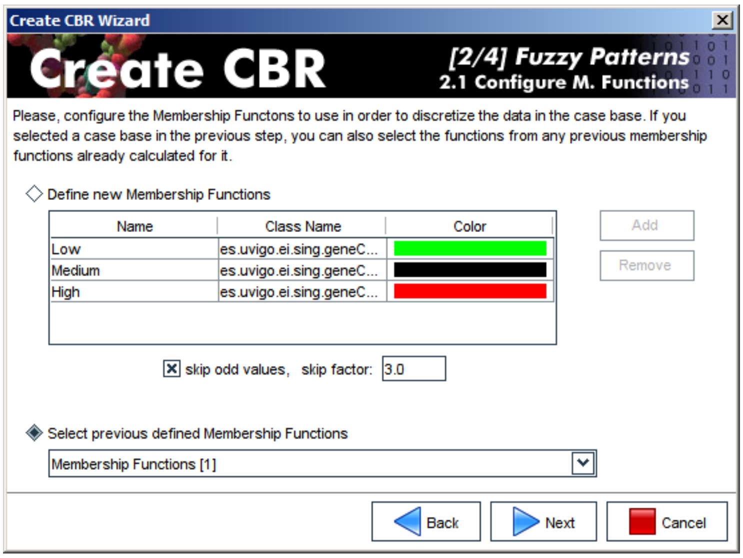

As in the previous case, in order to automate the fuzzy discretization process you can select a previously defined configuration or specify a new one.

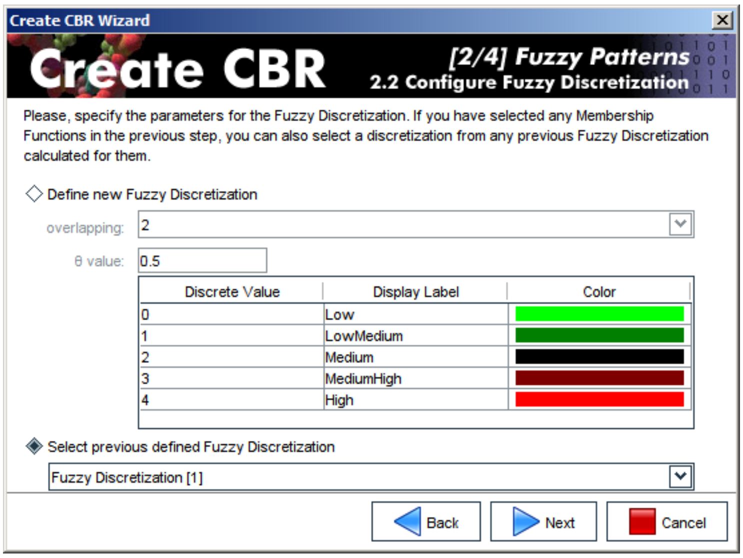

To configure the fuzzy patterns generation and the discriminant fuzzy pattern selection you have to provide a value for the π parameter.

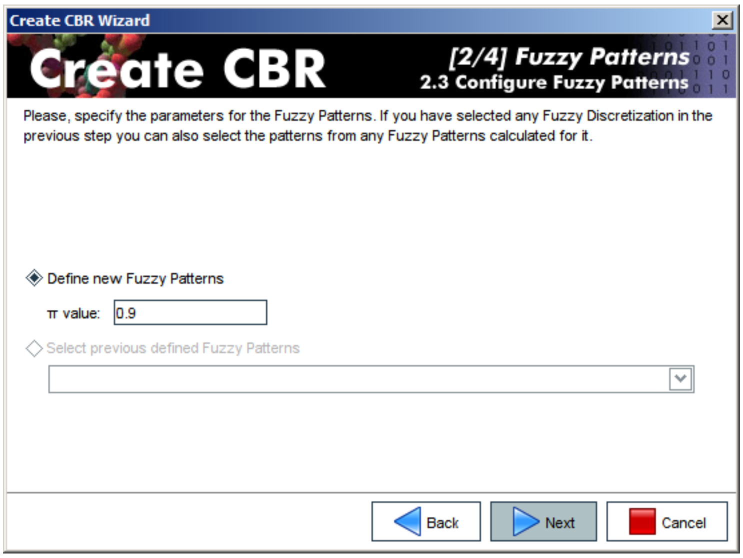

Once the DFP configuration is stored, you have to setup the parameters of the GCS network to use. As in previous case you can select a previously defined configuration or specify a new one.

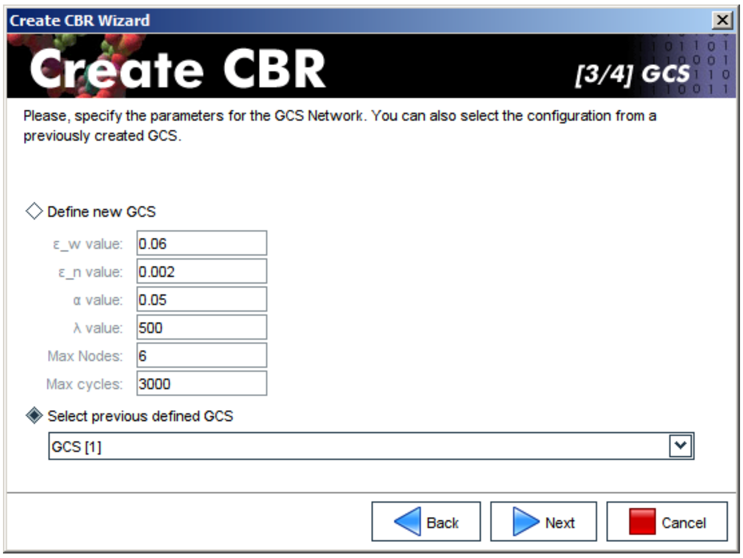

In the last step you have to specify a name for the CBR configuration file (in our example *Leukemia*) by pressing the `browse` button. GENECBR will automatically add the extension .cbr to this file (*Leukemia.cbr*) saving it in the <CBR_FILES> directory.

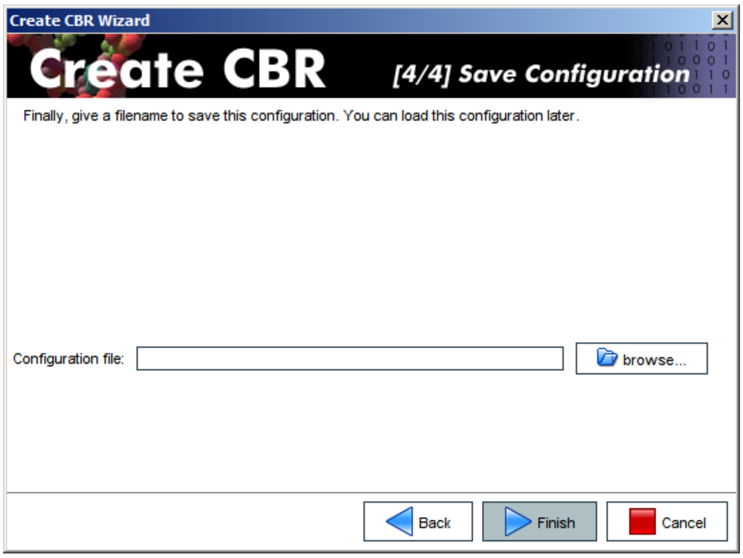

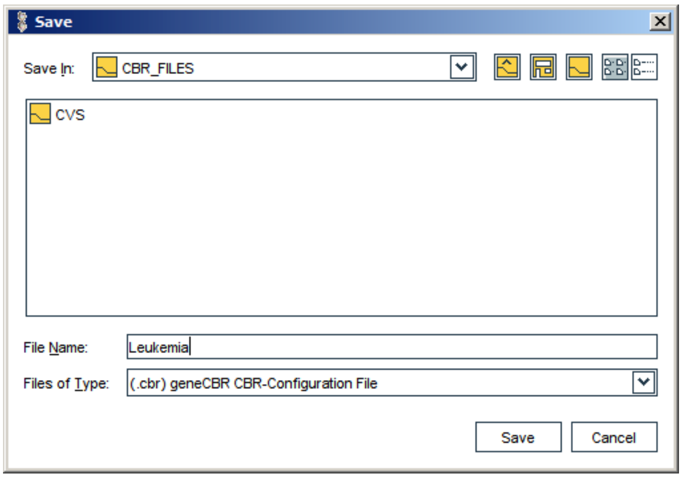

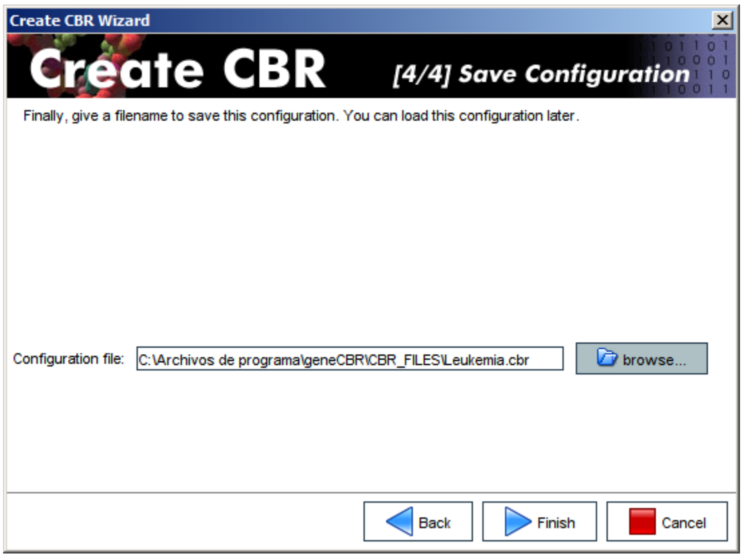

Once all the steps of the wizard are completed GENECBR starts to produce the required files for using the Diagnostic Mode. During this process you will see a progress dialog bar and then you will obtain a confirmation message.

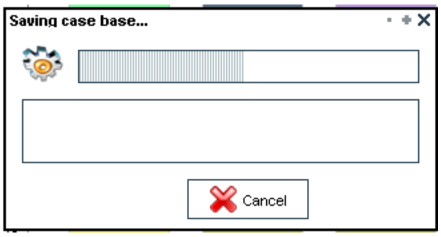

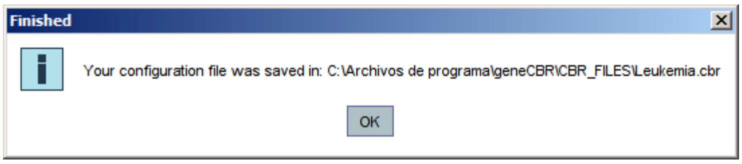

Finally, GENECBR gives you the option of executing the Diagnostic Mode to test this configuration.

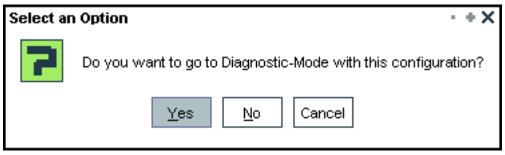

Load CBR

Note: This operation allows the expert to use preconfigured application able to automatically classify new incoming microarrays samples (with unknown class).

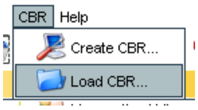

By executing the `CBR->Load CBR...` menu option, you can load a preconfigured GENECBR configuration to go to Diagnostic Mode.

In the file chooser dialog you have to specify a previously saved GENECBR configuration file.

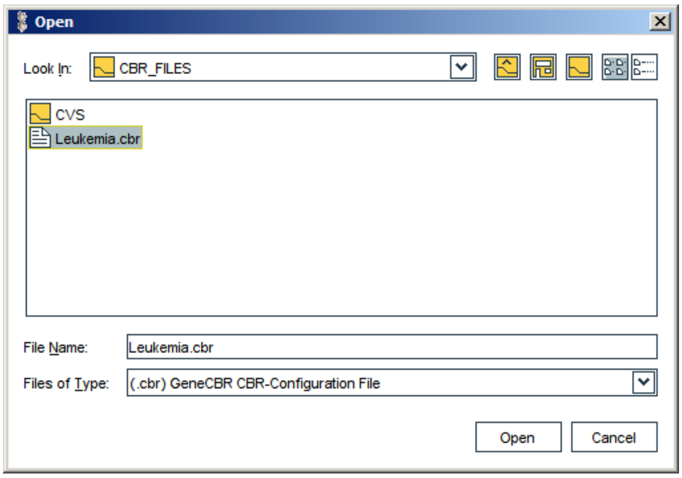

Help menu

Update NetExplorer Database

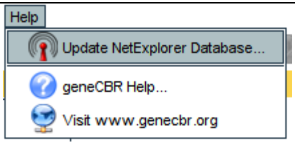

In order to maintain the `NetExplorer DB Query` advanced module up-to-date, GENECBR provides a free update service for downloading last minute information about gene annotations.

To execute this functionality you have to go to the `Help->Update NetExplorer Database...` menu option. During the on-line updating discovery process you will see a progress dialog bar.

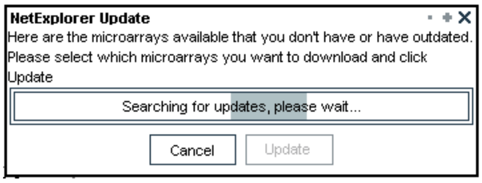

If no server is available for downloading the upgrades, an error message is displayed.

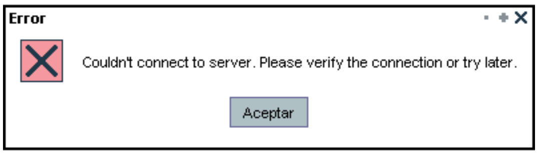

If you have all your files up-to-date and you do not need an actualization, the following informative message appears.

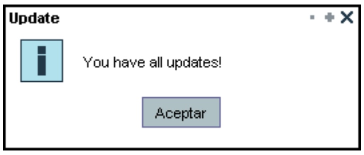

Otherwise, if some of your files are obsolete you will see an input dialog for selecting those files you want to download.

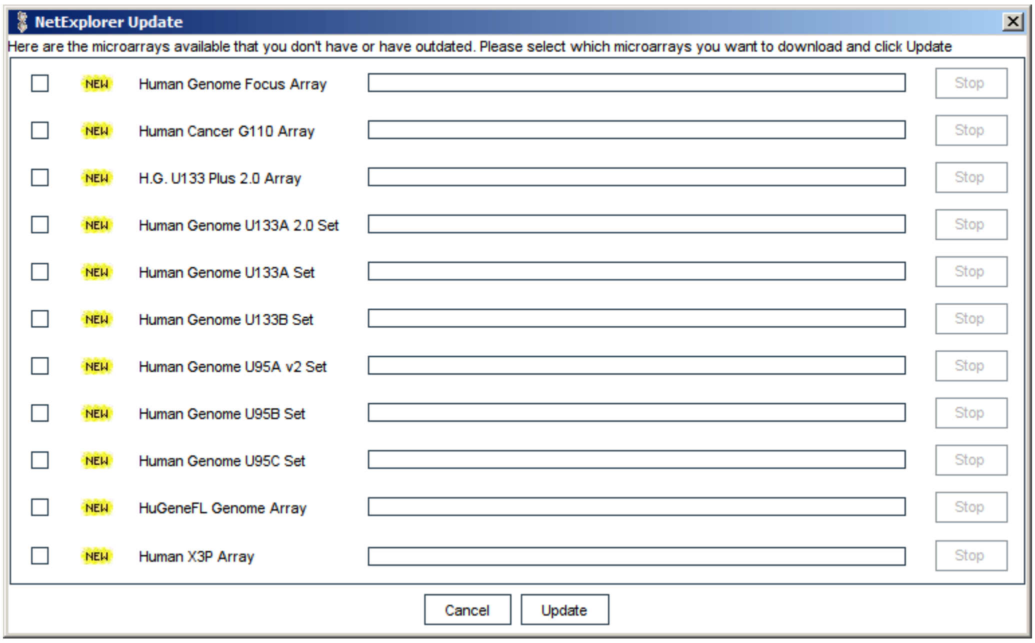

By pressing the `Update` button the process starts showing the progress of the operation.

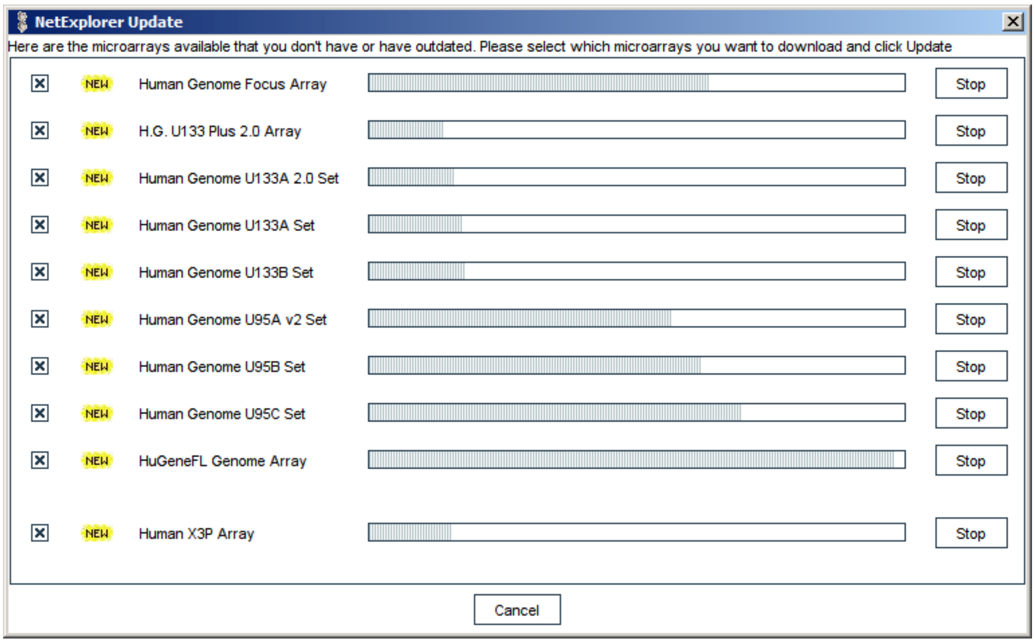

Once the update process has correctly finished, the following informative message appears.

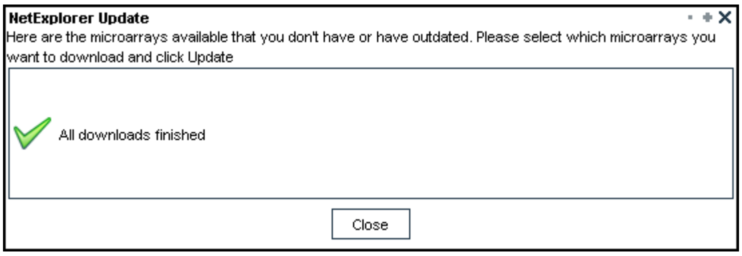

GENECBR Help

A detailed explanation about implemented options and configurable parameters in GENECBR is available from the `Help->geneCBR Help...` menu or by pressing de `F1` key.

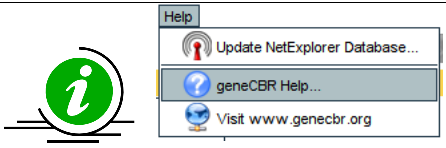

Basic information to help you get started with the application as well as detailed documentation can be accessed using the integrated on-line GENECBR help.

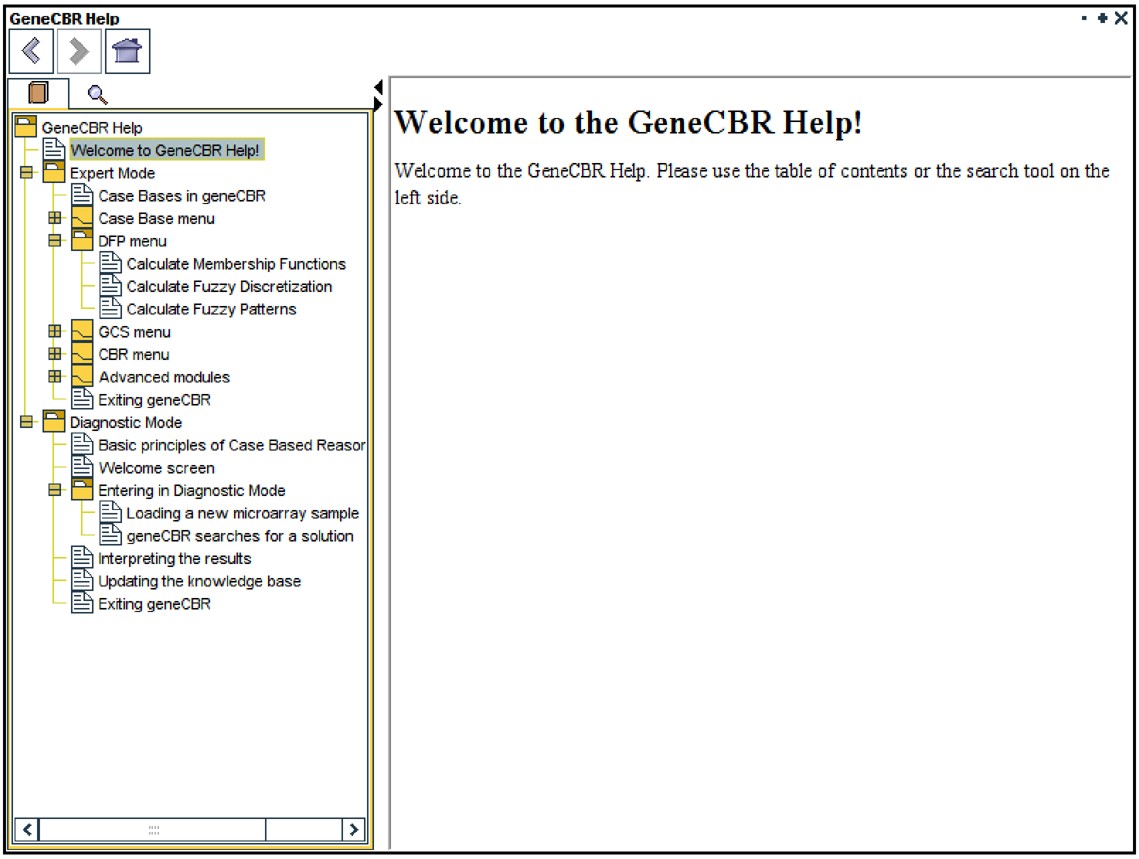

Moreover, in several operations executed by GENECBR the following fade tooltip briefly appears to guide the user to the recommended chapter in the help.

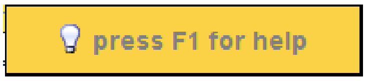

Visit www.genecbr.org

GENECBR portal on Internet is easily accessible through from the `Help->Visit www.genecbr.org` menu.

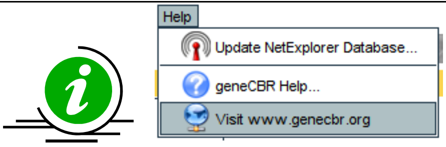

If you want to check the existence of news about the application and stay tuned for available updates, you should periodically check the GENECBR portal.

By selecting the `Help->Visit www.genecbr.org` menu option your default web browse will automatically load the GENECBR portal.

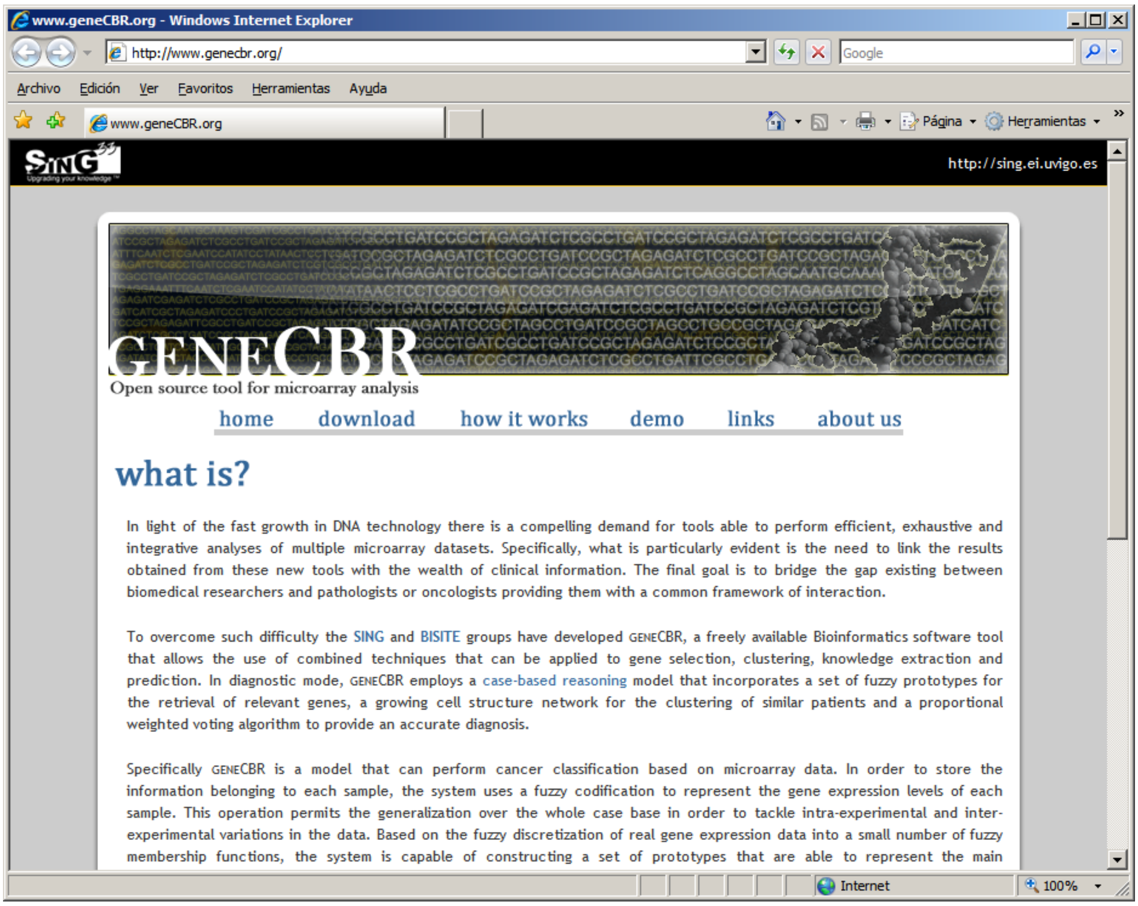

Advanced modules

Log module

Note: This panel gives the expert valuable information about all the actions executed in GENECBR Expert Mode.

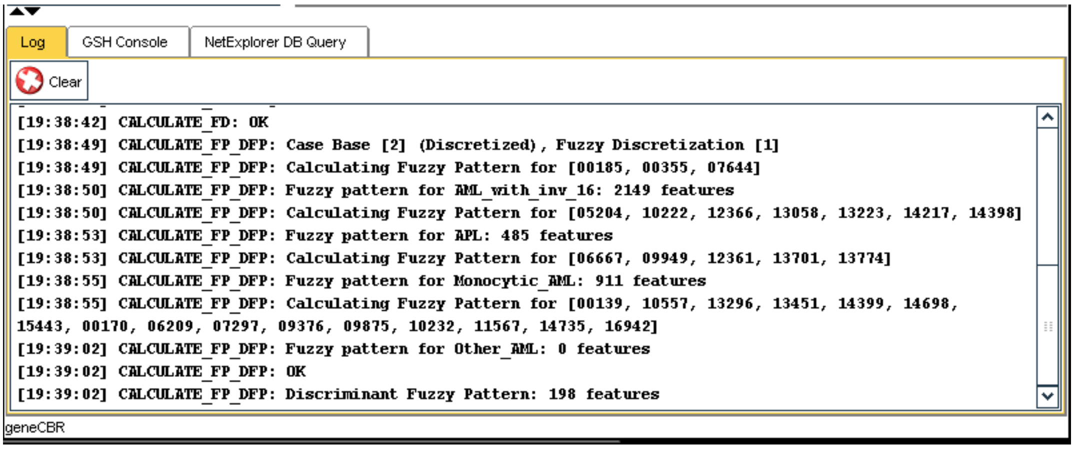

GSH Console

Note: This panel gives the programmer the possibility of changing and augmenting the functionality of GENECBR Expert Mode by executing scripts in an interactive way.

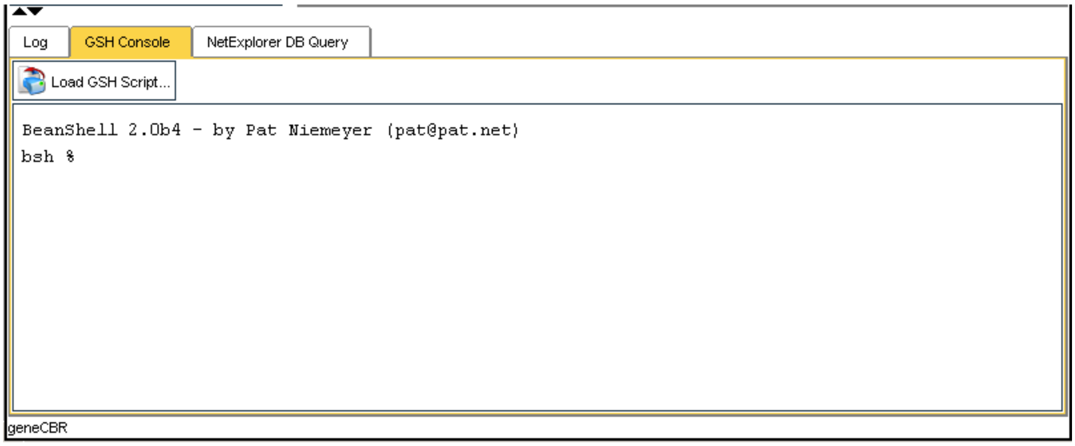

NetExplorer DB Query

Note: This panel allows the expert to perform integrated searches to locate relevant information

about selected genes.

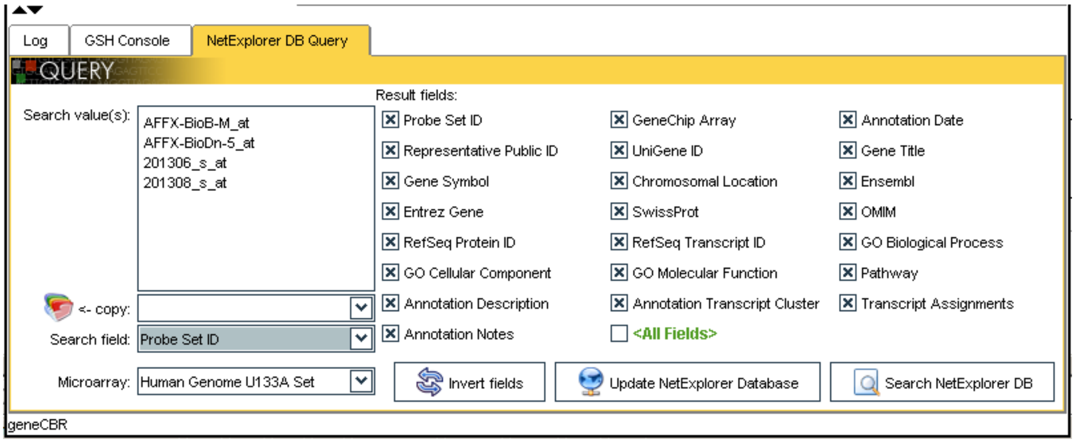

Exiting GENECBR

When you click in the right upper cross to close the GENECBR application, a confirmation message is showed in order to process your request.

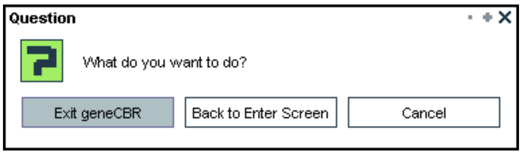

Bibliography

[1] F. Díaz, F. Fdez-Riverola, D. Glez-Pena, J.M. Corchado. Using Fuzzy Patterns for Gene Selection and Data Reduction on Microarray Data. 7th International Conference on Intelligent Data Engineering and Automated Learning: IDEAL 2006, (2006) pp. 1087-1094.

[2] F. Díaz, F. Fdez-Riverola, D. Glez-Pena, J.M. Corchado. Applying GCS Networks to Fuzzy Discretized Microarray Data for Tumour Diagnosis. 7th International Conference on Intelligent Data Engineering and Automated Learning: IDEAL 2006, (2006) pp. 1095-1102.

[3] F. Fdez-Riverola, F. Díaz, J.M. Corchado, J.M. Hernández, J. San Miguel: Improving Gene Selection in Microarray Data Analysis using Fuzzy Patterns inside a CBR System. Proceedings of the ICCBR 2005 Conference, (2005) 23-26.

[4] F. Díaz, F. Fdez-Riverola, J.M. Corchado: GENE-CBR: a Case-Based Reasoning Tool for Cancer Diagnosis using Microarray Datasets. Computational Intelligence (2006) 22(3-4):254-268.

[5] D. Glez-Peña, F. Díaz, F. Fdez-Riverola, J.R. Méndez, J.M. Corchado. Fuzzy Patterns and GCS Networks to Clustering Gene Expression Data. Fuzzy Systems in Bioinformatics, Bioengineering and Computational Biology. Springer (2008).
