# Supplementary material for: geneCBR: a translational tool for multiple-microarray analysis and integrative information retrieval for aiding diagnosis in cancer research
Source: BMC Bioinformatics. 2009 Jun 18;10:187. doi: 10.1186/1471-2105-10-187 (PMC2703634; doi:10.1186/1471-2105-10-187)
Supplement: Additional file 2 — Diagnostic Mode manual. This document will guide the user through a step by step tutorial showing the capabilities of geneCBR to automatically classify new microarray samples in Diagnostic Mode. [file 1471-2105-10-187-S2.pdf]

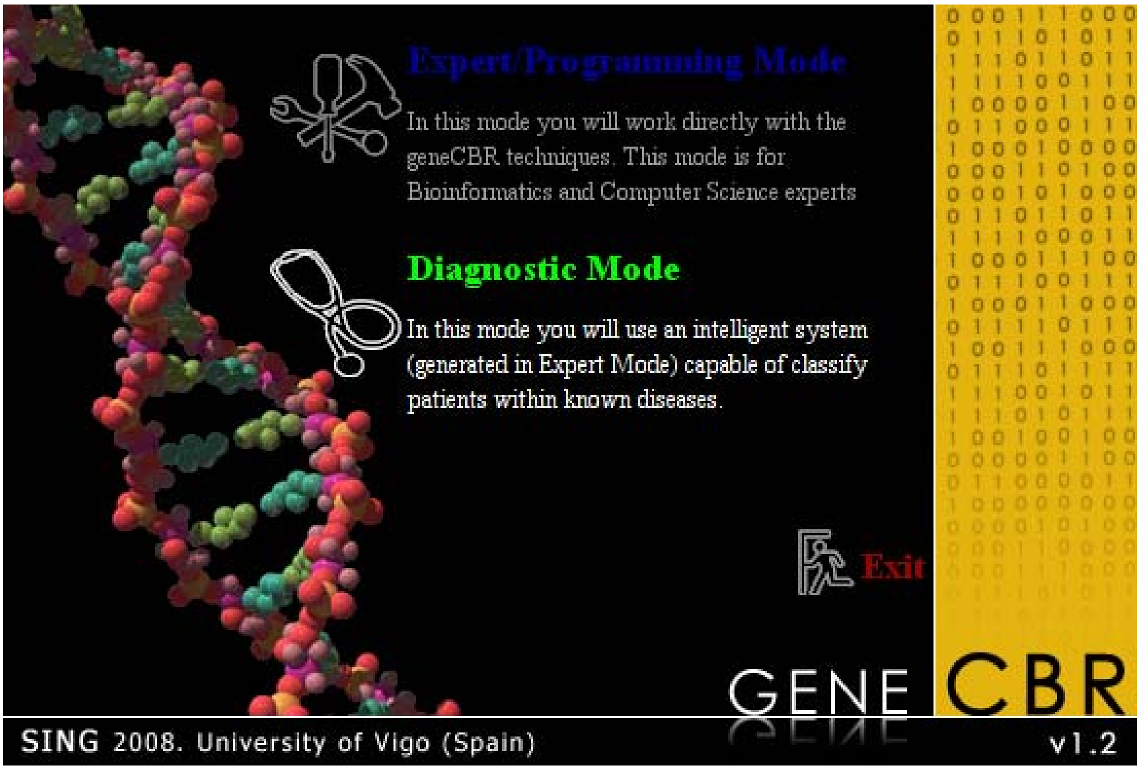

GENECBR

Diagnostic Mode Manual

This document will guide you through a step by step tutorial showing the capabilities of GENECBR to automatically classify new microarray samples in Diagnostic Mode.

Contents

Basic principles of Case Based Reasoning applications 1
Welcome screen.. 3
Entering in Diagnostic Mode.. 4
    Loading a new microarray sample.. 4
    GENECBR searches for a solution.. 5
Interpreting the results... 6
Updating the knowledge base.. 8
Exiting GENECBR... 8

Basic principles of Case Based Reasoning applications

GENECBR is constructed following the design principles of a Case Based Reasoning (CBR) application.

Case-based reasoning is a computational reasoning paradigm that involves the storage and retrieval of past experiences to solve new problems. An advantage of CBR systems as a problem-solving paradigm is that it is applicable to a wide range of problems, and is particularly relevant in scientific domains, where there is a wealth of data but often a lack of theories or general principles.

A case-based reasoning system solves new problems by adapting solutions that were used to solve previous problems. The case base holds a number of cases, each of which represents a problem together with its corresponding solution. Once a new problem arises, a possible solution to it is obtained by retrieving similar cases from the case base and studying their recorded solutions. A CBR system is dynamic in the sense that, in operation, cases representing new problems together with their solutions are added to the case base, redundant cases are eliminated and others are created by combining existing cases.

A CBR system analyses a new problem situation, and by means of indexing algorithms, retrieves previously stored cases together with their solution by matching them against the new problem situation, then adapts them to provide a solution to the new problem by reusing knowledge stored in the form of cases, in the case base. All of these actions are self-contained and may be represented by a cyclic sequence of processes, in which human interaction may be needed. Case-based reasoning can be used by itself or as part of another intelligent or conventional computing system. Furthermore, case-based reasoning can be a particularly appropriate problem-solving strategy when the knowledge required to formulate a rule-based model of the domain is difficult to obtain, or when the number or complexity of rules relating to the problem domain is too great for conventional knowledge acquisition methods.

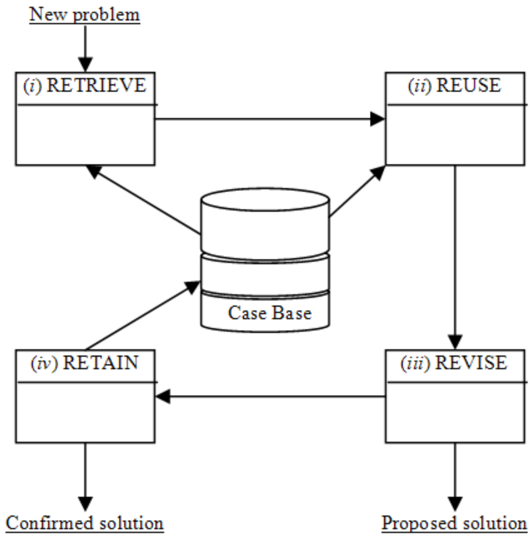

(i) ***Retrieve*** the most relevant case(s).

(ii) ***Reuse*** the case(s) to attempt to resolve the problem.

(iii) ***Revise*** the proposed solution if necessary.

(iv) ***Retain*** the new solution as a part of a new case.

A typical CBR system is composed of four sequential steps which are called into action each time a new problem is to be solved. The following figure outlines the basic CBR cycle.

The purpose of the retrieval step is to search the case base and select one or more previous cases that most closely match the new problem situation, together with their solutions. The selected cases are reused to generate a solution appropriate to the current problem situation. This solution is revised if necessary and finally, the new case (i.e. the problem description together with the obtained solution) is stored in the case base. Cases may be deleted if they are found to produce inaccurate solutions, they may be merged together to create more generalized solutions, and they may be modified, over time, through the experience gained in producing improved solutions. If an attempt to solve a problem fails and it is possible to identify the reason for the failure, then this information should also be stored in order to avoid the same mistake in the future. This corresponds to a common learning strategy employed in human problem-solving. Rather than creating general relationships between problem descriptors and conclusions, as is the case with rule-based reasoning, or relying on general knowledge of the problem domain, CBR systems are able to utilize the specific knowledge of previously experienced, in the form of concrete problem situations. A CBR system provides an incremental learning process because each time a problem is solved, a new experience is retained, thus making it available for future reuse.

In the CBR cycle there is normally some human interaction. Whilst case retrieval and reuse may be automated, case revision and retention are often undertaken by human experts.

Welcome screen

The first step needed to classify a new microarray sample is to load a previously saved CBR configuration file (see the Expert Mode Manual).

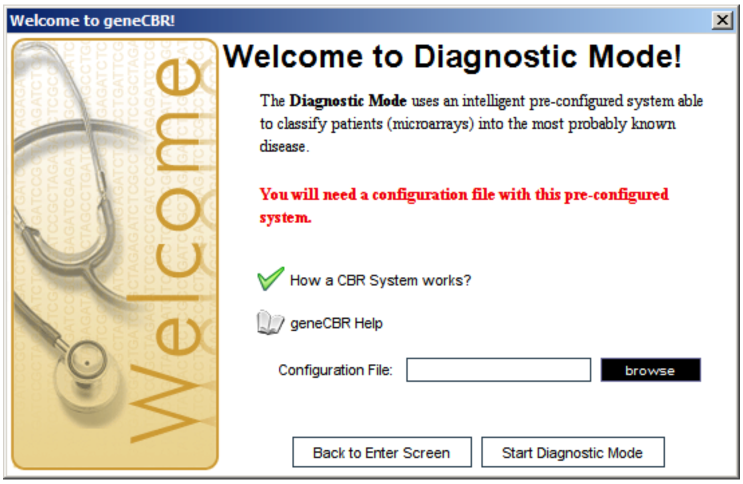

In the welcome screen this can be done by specifying a file with .cbr extension (in our example *Leukemia.cbr*) and pressing the `Start Diagnostic Mode` button.

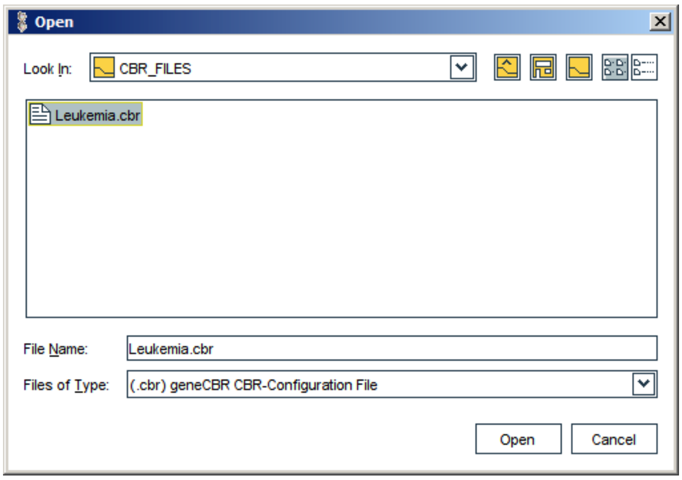

Next, you will see a progress dialog bar meanwhile the case base is loaded.

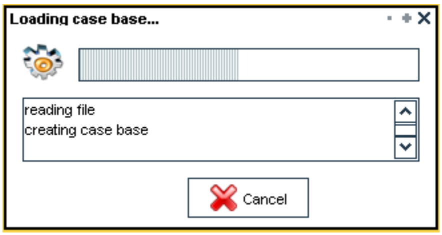

Entering in Diagnostic Mode

When GENECBR is ready to use, a simple interface appears where the case base containing all the available patients and their meta-data information are showed to you.

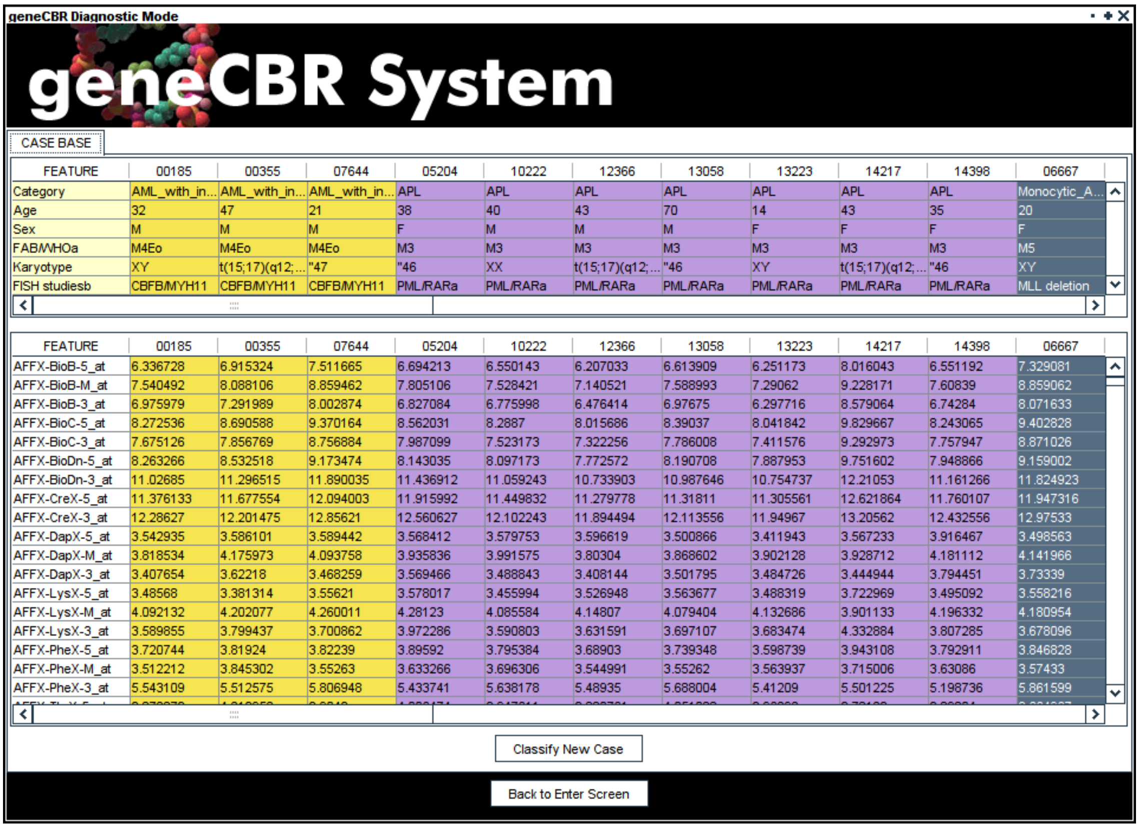

From this screen you can go back to the enter screen or classify a new microarray sample by pressing the `Classify New Case` button.

Loading a new microarray sample

To load the raw data belonging to a new (unclassified) microarray sample, you have to select a text-based, comma-separated file in the file chooser dialog (see the Expert Mode Manual for more information about the specific format).

In our example we load the file *Leukemia_test_01.csv* containing one microarray sample of type APL (not present in the train case base).

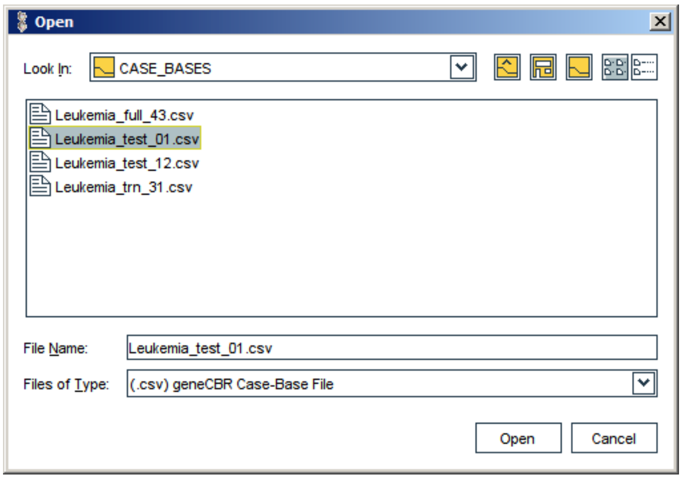

GENECBR searches for a solution

Once the new microarray sample is validated, GENECBR evolves through the 4-step process in order to find the best classification for this patient.

The first step (RETRIEVE) involves the execution of the DFP algorithm (explained in the Expert Mode Manual) for the selection of the most relevant genes. During this process, you will see a progress dialog bar showing related information about the actions who take place.

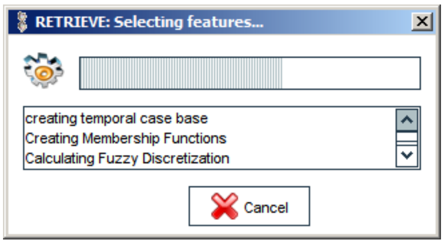

The second step (REUSE) involves the training of the GCS network in order to search for those patients most similar to the new microarray sample. As is the previous case, you will see a progress dialog bar meanwhile this process is executed.

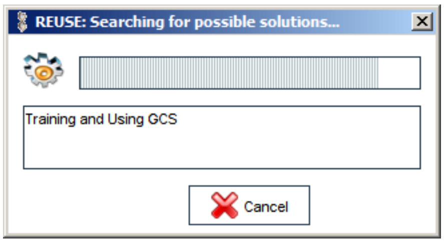

Once the main reasoning cycle is terminated (RETRIEVE & REUSE steps), GENECBR shows you the outcome of the classification process. As a result, three new tabs are available in the application.

From the REVISE tab, it can be seen how the new microarray sample is proposed to be classified as APL. In this tab, GENECBR also shows all the patients belonging to the same node of the trained clustering network.

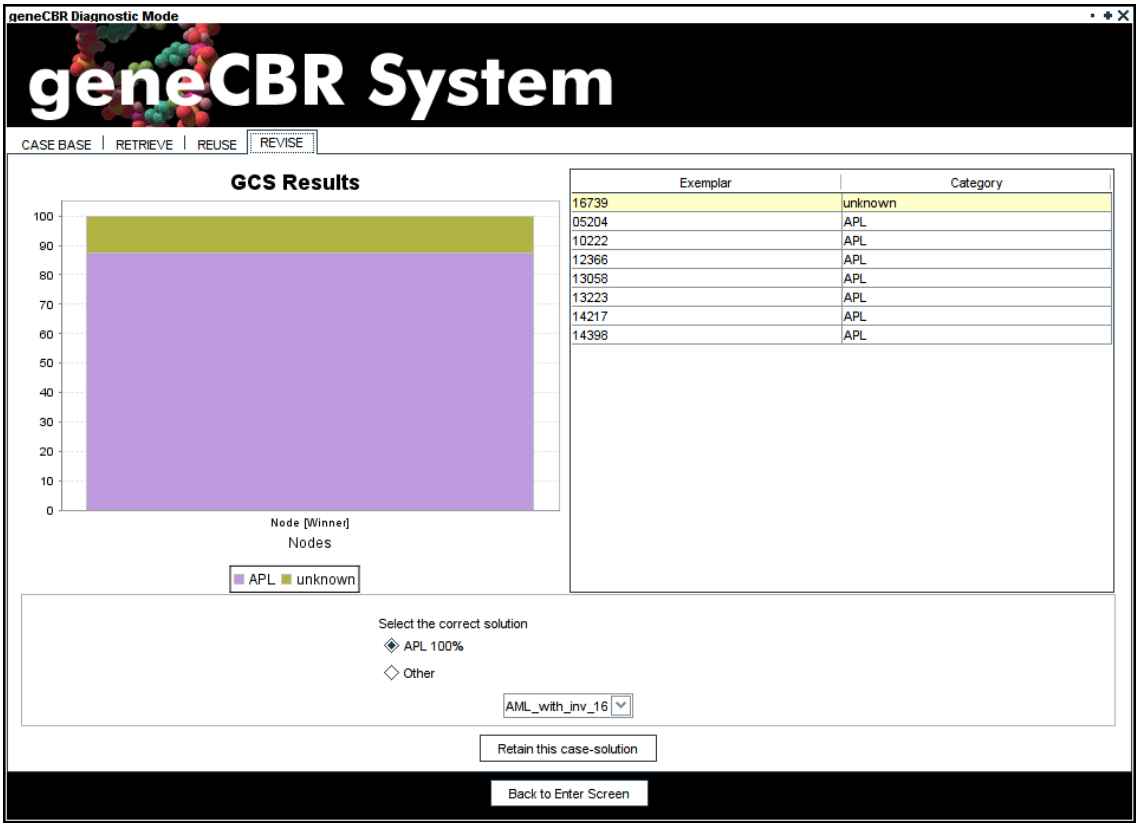

In order to find additional clues about the decision adopted by the GENECBR system, you can inspect the RETRIEVE and REUSE tabs.

Interpreting the results

Every time a new classification is proposed by the system, GENECBR shows the partial results achieved in all the reasoning cycle.

From the RETRIEVE tab, you can examine the genes belonging to the final discriminant fuzzy pattern taken into consideration during the classification process. In the upper part of the screen a brief summary is showed containing information about the different linguistic labels assigned to the different fuzzy patterns. From the lower part of the screen, you can explore the value of the assigned label to any gene.

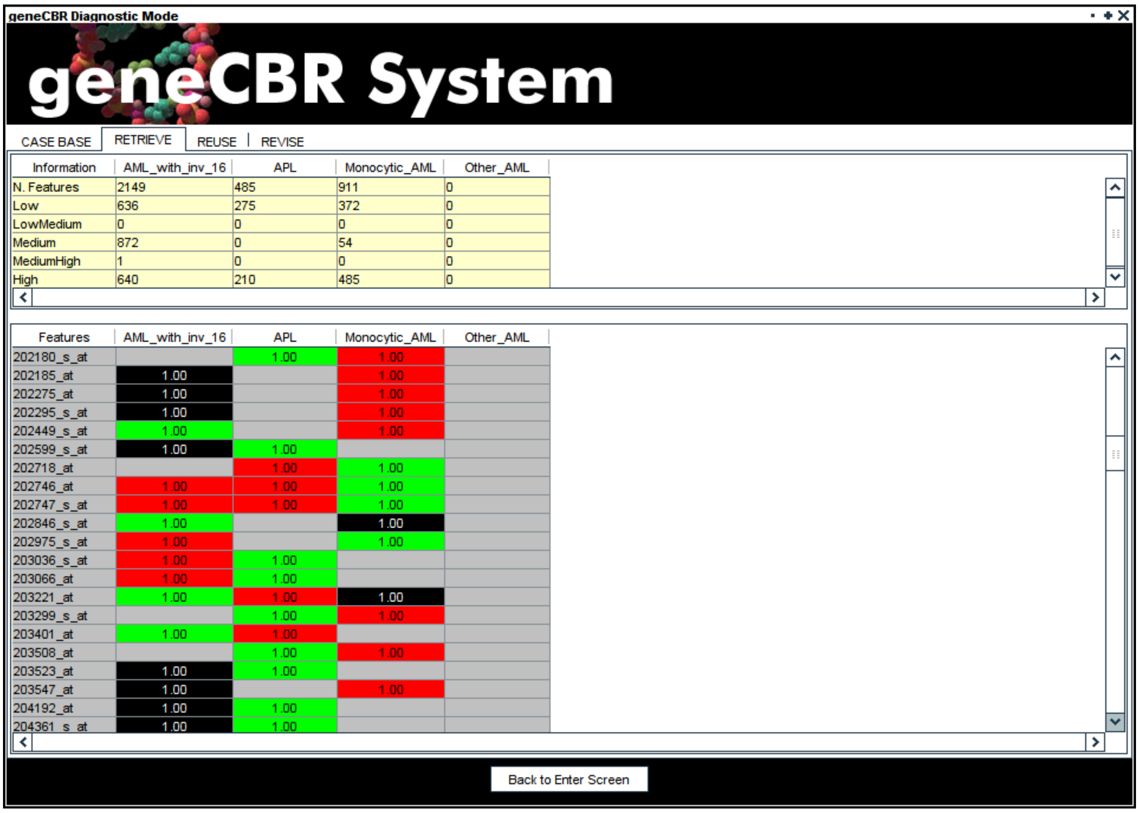

From the REUSE tab in the upper part, you can review all the nodes constructed by the clustering network. In our example, the node assigned to the new microarray sample was *Node2* (the node containing the *unknown* sample). Moreover, in the lower part of the REUSE tab, you can inspect all the patients belonging to each node.

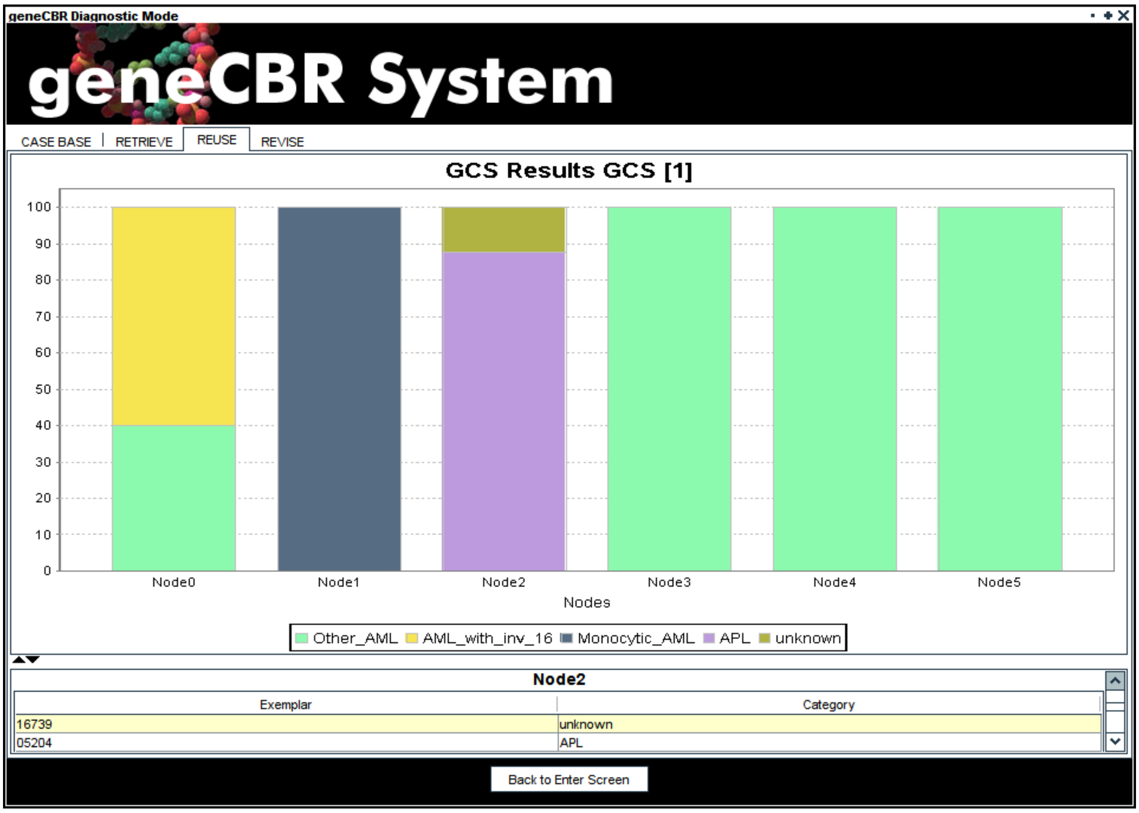

Updating the knowledge base

Once the user selects a category for the new microarray sample and presses the `Retain this case-solution` button, the system evolves through the RETAIN step. During this process, GENECBR stores the new case in its case base for future reuse. A progress dialog bar accompanies this step.

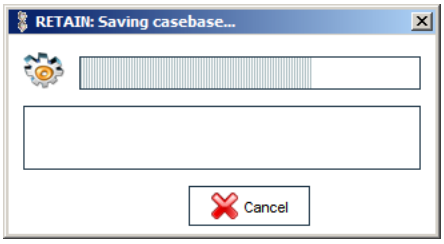

When the last step finishes, GENECBR shows the `RETAIN` tab where the new microarray sample (patient ID 16739) is added to the case base.

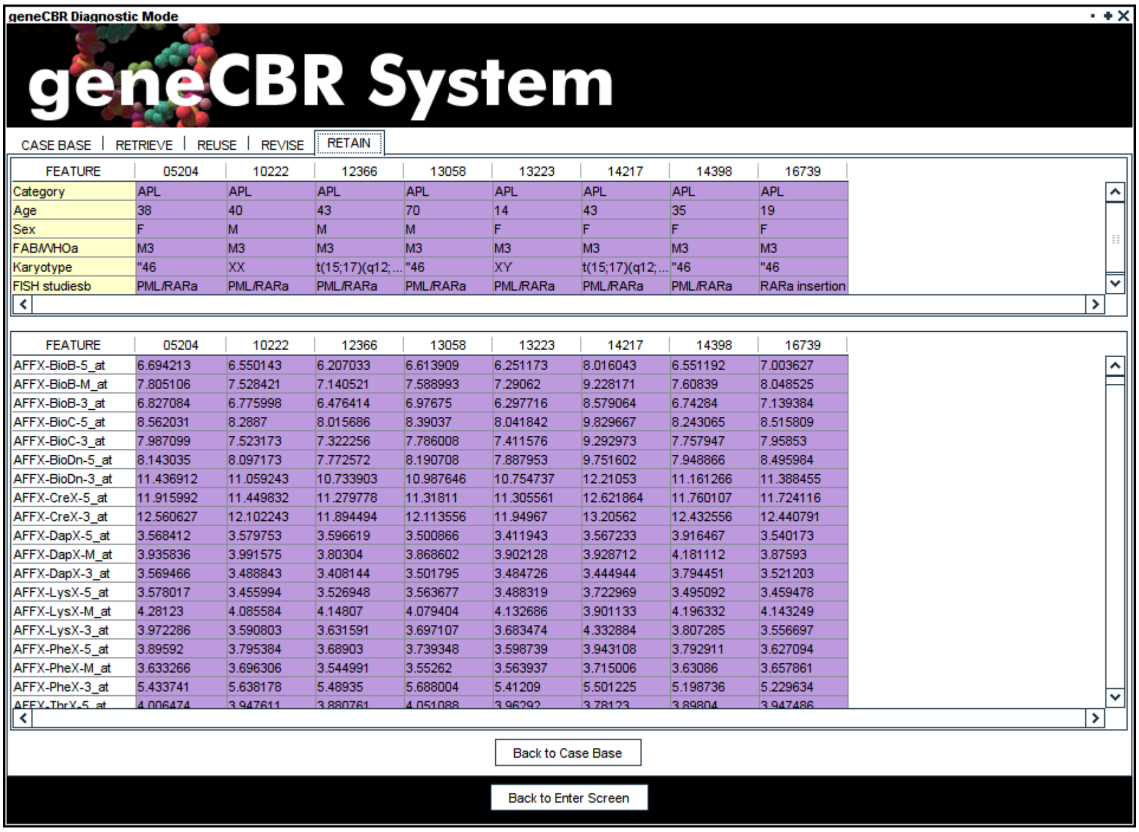

From this screen you can go back to the enter screen or repeat the classification process by pressing the `Back to Case Base` button.

Exiting GENECBR

When you press the `Back to Enter Screen` button a confirmation message is showed in order to process your request.

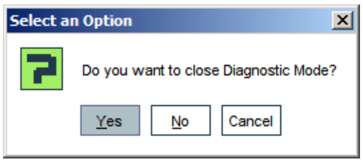
Select an Option
Do you want to close Diagnostic Mode?
Yes
No
Cancel
