# Supplementary material for: geneCBR: a translational tool for multiple-microarray analysis and integrative information retrieval for aiding diagnosis in cancer research
Source: BMC Bioinformatics. 2009 Jun 18;10:187. doi: 10.1186/1471-2105-10-187 (PMC2703634; doi:10.1186/1471-2105-10-187)

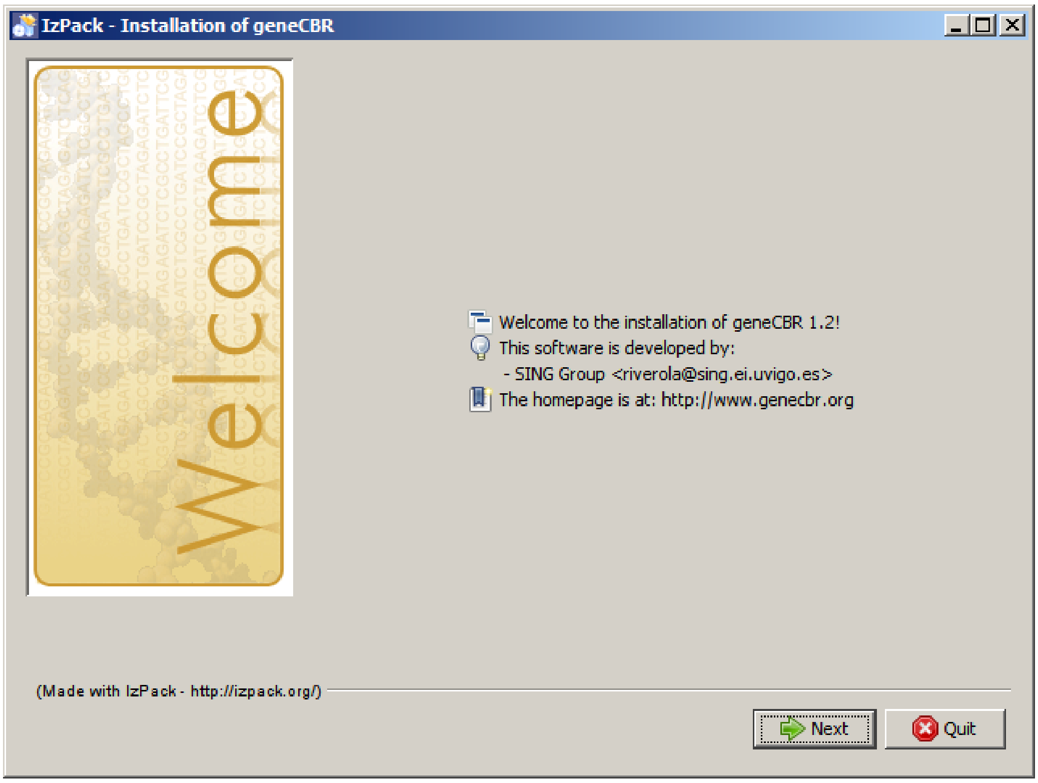

GENECBR

Installation Guide

This manual covers the installation of GENECBR *and its related components through the utilization of our installation wizard.*

Contents

Obtaining and executing the installation wizard 1
Specifying the installation directory 2
Selecting installation packages 2
Copying program files 3
Defining setup shortcuts 3
Finalizing the installation 4
Uninstaling geneCBR 4

Obtaining and executing the installation wizard

GENECBR is distributed with an installation wizard that will guide you through the installation process. You can download the required files from the “*download*” section of the GENECBR portal (http://www.genecbr.org/download.htm).

Depending on your Operating System you will have to download different files.

	**Windows users**	`setup-geneCBR.zip`

Decompress the zip file, execute `setup.exe` and it will launch the installation wizard. If you do not have a Java Runtime Environment (JRE) installed in your computer, the installation wizard will install first this component, and then it will continue with the GENECBR installation. Follow the instructions on the screen to complete the installation.

	**Linux / MAC users**	`setup-geneCBR.jar`

Double click on `setup-geneCBR.jar` file and it will launch the installation wizard. If you do not have a Java Runtime Environment (JRE) installed in your computer, first you need to download and install it from “*other downloads*” section of the GENECBR portal. Follow the instructions on the screen to complete the installation.

Specifying the installation directory

In this step you will be asked to provide the directory where the application will copy the program files.

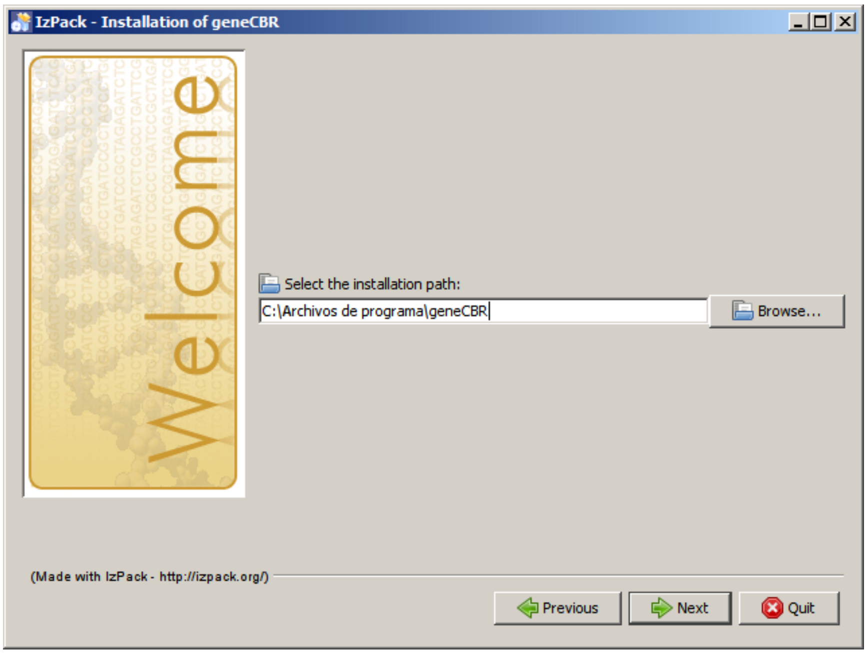

If you specify a non existing directory, the following message appears in order to confirm your selection.

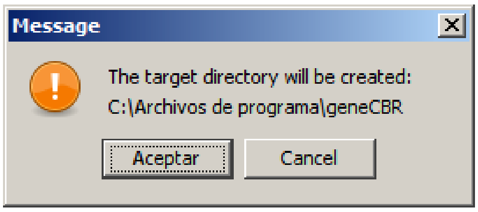

Selecting installation packages

In this screen you will be asked to specify the packages you want to install. By default, all packages are selected. The `Base` package is mandatory in order to get GENECBR working properly.

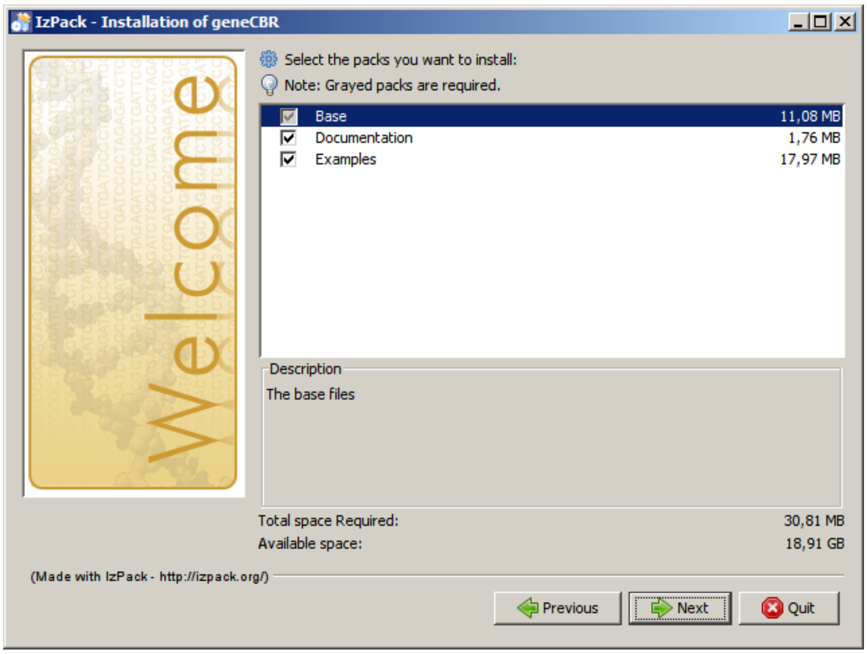

Copying program files

Once the installation wizard has all the necessary information for installing GENECBR, it starts copying the required program files to the hard disk of your computer. During the installation process a progress dialog is showed to the user.

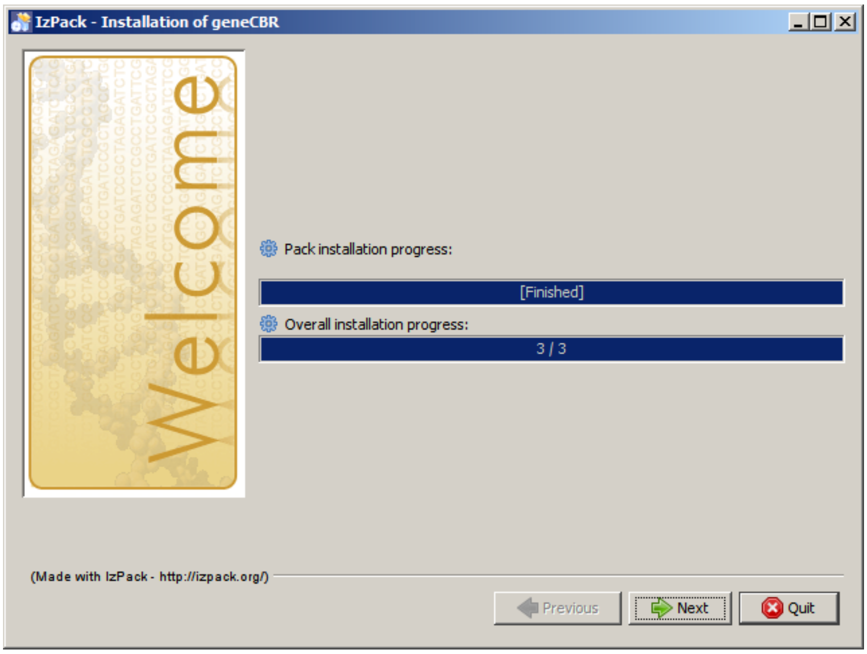

Defining setup shortcuts

In this screen you will be able to specify the desired shortcuts for the GENECBR application. If you want an additional icon on the desktop for launching GENECBR you have to check the corresponding option.

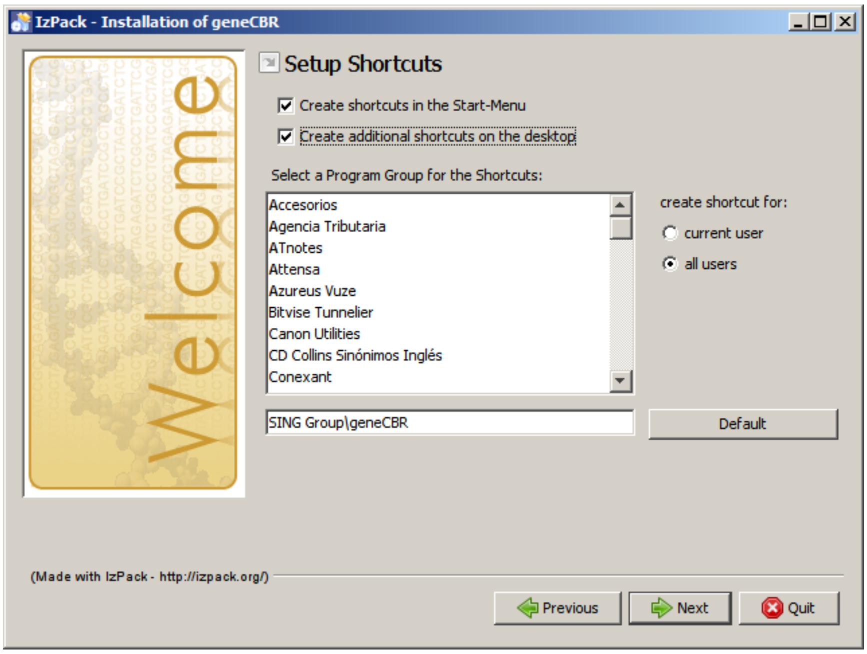

Finalizing the installation

Once the installation process finishes, it is showed to the user a final summary screen.

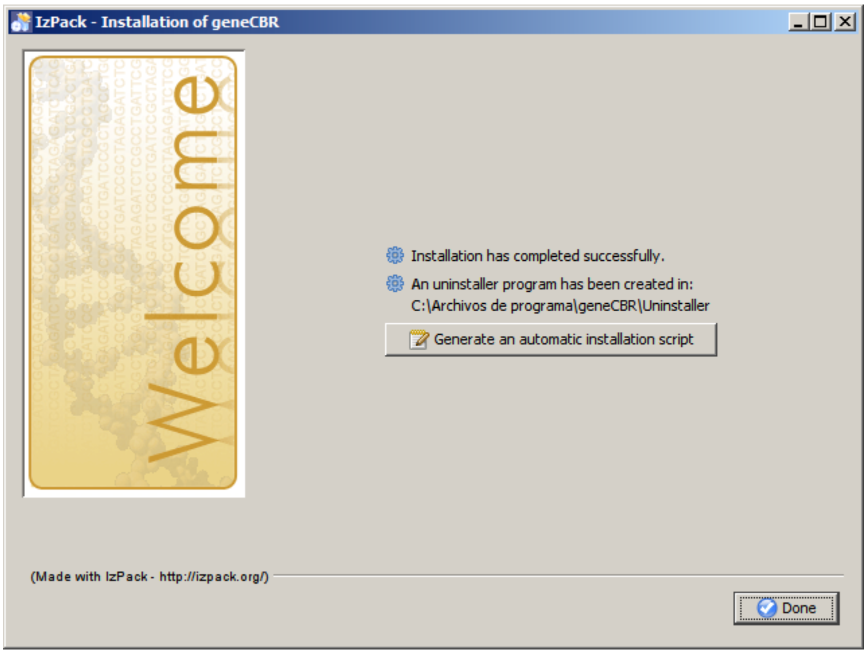

Uninstaling GENECBR

In order to desinstall GENECBR you can execute the existing shortcut available in the Start-Menu. If you want to delete all the existing files below the installation directory, you have to check the corresponding option.

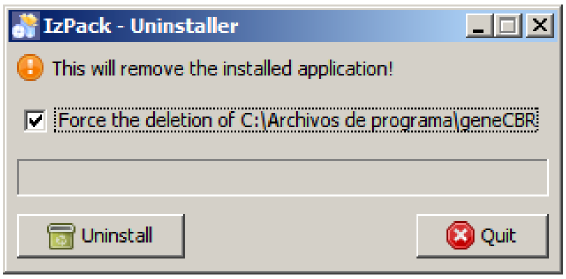

Once the uninstalling procedure finishes, it is showed to the user a final summary screen.

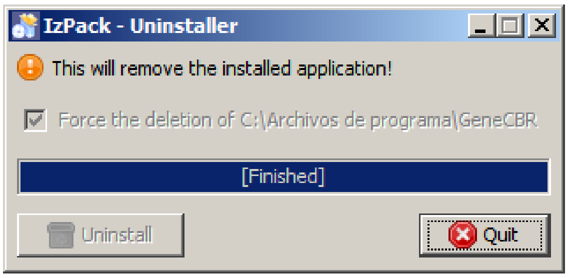

Supplement: Additional file 3 — Installation guide. This manual covers the installation of geneCBR and its related components through the utilization of an installation wizard. [file 1471-2105-10-187-S3.pdf]
